# RNA m5C methylation orchestrates BLCA progression via macrophage reprogramming

**DOI:** 10.1111/jcmm.17826

**Published:** 2023-07-05

**Authors:** Dali Yan, Yongsong Xie, Liyuan Huang, Yi Zhang, Runhuan Gu, Huaibing Xie, Xing Huang, Hao Luo

**Affiliations:** ^1^ Department of Oncology The Affiliated Huai'an Hospital of Xuzhou Medical University and the Second People's Hospital of Huai'an Huai'an China; ^2^ Department of Geriatrics The Third Hospital of Kunshan City Kunshan China; ^3^ Department of Urology The Affiliated Huai'an Hospital of Xuzhou Medical University and the Second People's Hospital of Huai'an Huai'an China; ^4^ Department of Pathology Jiangsu Cancer Hospital, Jiangsu Institute of Cancer Research, Nanjing Medical University Affiliated Cancer Hospital Nanjing China; ^5^ Department of Oncology Lian Shui People's Hospital Affiliated to Kangda College of Nanjing Medical University Huai'an China

## Abstract

Recently, epigenetics showed essential roles in tumour microenvironment (TME) and immunotherapy response, however, the functions of RNA 5‐methylcytosine (m5C) modification in TME remains unknown. According to 13 m5C regulators, we evaluated 412 BLCA patients from The Cancer Genome Atlas (TCGA) database. The m5C score was constructed by unsupervised clustering analysis and principal component analysis (PCA) algorithms. Gene set variation analysis (GSVA), ESTIMATE algorithm, and immunohistochemical (IHC) staining were performed. Macrophage chemotaxis assay was used to assess the M2 macrophages. Among the 412 patients, the frequency of mutation was 13%. m5C regulators was expressed significantly in BLCA tissue compared with normal tissue. Then, two m5C methylation modification patterns were identified with dissimilar TME cell infiltration patterns. The C1 alteration pattern in the m5C cluster was connected with better survival. In addition, we found that NSUN6 was highly correlated with recruitment of macrophages via bioinformatics and IHC. Further experiment validated that NSUN6 promoted HDAC10 expression by mediating m5C methylation, inhibited the transcription of macrophage‐associated chemokines and thus inhibited the recruitment of M2 macrophages. The m5C score constructed by m5C modification pattern showed that high m5C score group had a better prognosis. This study uncovered the significant roles of m5C modifications in modulating the TME and indicated that NSUN6 could inhibit the recruitment of M2 macrophages via m5C methylation, which provided novel insight into epigenetic regulation of TME and clinical suggestions for immunotherapeutic strategies.

## 1 INTRODUCTION

Recently, the role of RNA modifications draws widespread attention in carcinogenic processing.^1^ Base on the RNA modification database, more than 150 RNA modifications were identified as the third epigenetics layer, which includes N6‐methyladenosine (m6A) and 5‐methylcytosine (m5C).^2^ N6‐methyladenosine (m6A) was recognized as a reversible mRNA modification which played important roles in various tumorigenesis, such as lung cancer, hepatocellular carcinoma and bladder cancer.(BLCA)^3^, ^4^ While m5C modification can regulate various RNA processing, such as RNA metabolic processes, RNA translation and export, alternative splicing, RNA stability and localization.^5^ Different from extensive studies about the function of DNA m5C modification in carcinogenesis, few are reported about that of RNA m5C modification. Same as m6A, the m5C methylation was altered by ‘writers’ (methyltransferases), ‘erasers’ (demethylases) and ‘readers’.^6^ The m5C methyltransferases includes NSUN1‐7 and DNMT1‐3, while TET2 acts as ‘erasers’ to remove the methyl group. In addition, ALYREF and YBX1 act as m5C ‘readers’ in mammals to bind the m5C motif. According to previous reports, the m5C modification was primarily enriched in guanine‐cytosine (GC)‐rich regions closed to 3′UTR and 5′UTR.^5^ There are over 10,000 potential m5C modification sites detecting in the whole human transcriptome.

BLCA, a complex and heterogenetic disease, is one of the top 10 common tumours around the world.^7^ The carcinogenesis of BLCA is multifactor and multiply processes, such as internal genetic factors of tumour cells and external micro‐environmental factors.^8^ TME contains various stromal cells including macrophages, fibroblasts and immune cells. The complexity and diversity of TME are identified to result into immune escape and drive the progression of cancer.^9^ Then, the infiltration characterisation of TME cells make significant impact on the efficacy of immunotherapy.^10^ Thus, it is important to landscape the complexity and diversity of TME in epigenetic and genetic levels and identify the various immune phenotypes, which may guide the strategies of immunotherapy. Alteration of epigenetic level makes great effort to TME. Several studies have demonstrated the relevance between m6A and TME.^11^, ^12^ While the relevance between TME infiltrating immunocytes and m5C modification recently draws great attention.^6^ TET family, one of m5C demethylases, was associated with activating dendritic cells and triggering regulatory T (Treg) cells.^13^ In addition, hypermethylated m5C sites hold the stability of numerous oncogene RNAs to facilitate the pathogenesis of bladder tumour via genomic variation and the dis‐regulation of m5C regulators.^14^ Furthermore, the potential crosstalk of m5C regulators mediating TME infiltration characterisation was detected. Li et al. revealed that epigenetic phenotypes were identified by m5C modification.^7^ Despite the discovery of potential phenotypes and prognostic biomarkers, further understanding of molecular mechanisms and potential cross‐talk pathways remains uncovered. Thus, comprehensive understanding the relationship between m5C regulators and the TME cell infiltration is of great significance.

Here, CNV and somatic mutation were summarized upon 13 m5C genes in 412 BLCA patients. And three m5C modification patterns were integrated according to the characteristics of TME cell infiltration and prognosis. Besides, we found that NSUN6 could inhibit the recruitment of M2‐type macrophages and suppress macrophage‐associated chemokine expression via regulating the histone deacetylase family. These results provide a reference for improving the treatment of BLCA.

## 2 METHODS

### 2.1 Data collection and preprocessing

m5C systematic search of the cancer genome atlas (TCGA) dataset for BLCA was conducted. TCGA BLCA gene expression RNA‐seq of FPKM, copy number variation (CNV) (GISTIC Annotation), Masked Somatic Mutation (VarScan2 Annotation), and associated clinical data were also downloaded through UCSC‐Xena (http://xena.ucsc.edu/).

11 m5C related regulators had expression in TCGA datasets for BLCA analysis were extracted. (We detected 13 m5C modification‐related genes.) These 13 m5C regulators are composed of 11 writers (DNMT1, DNMT2, DNMT3B, DNMT3A, NSUN7, NSUN6, NSUN5, NSUN4, NSUN3, NSUN2, NSUN1), one reader (ALYREF) and one eraser (TET2). Unsupervised clustering analysis was utilized to identify m5C modification patterns and categorize patients. By using non‐negative matrix factorization (NMF), the 11 m5C regulators were grouped with the BLCA tumour sample. The standard ‘Brunet’ was used for Non‐negative Matrix Factorization (NMF) analysis, which underwent 50 iterations. ‘NMF’ (R package) was selected to identify the average contour width of the common member matrix, setting the minimum members of each subclass to 10, and we set the clusters number from 2 to 10. And the optimal clustering number was performed as 2 according to the cophenetic distribution.

### 2.2 Functional enrichment analysis and GSVA(gene set variation analysis) analysis

GSVA analysis was conducted by GSVA(R package).^15^ The KEGG gene set was downloaded in https://www.kegg.jp/. In samples from expression dataset, the variations in route and biological process activity were assessed by GSVA. Using the R program limma, differential paths were identified when |t| >6.

### 2.3 TME cell infiltration estimation

The composition and TME in the two m5C‐modified patterns were analysed by estimating the relative RNA transcripts (TIMER2.0‐http://timer.cistrome.org/)^16^ method. R was employed to examine the connection between m5C regulators and several TME cell. We also quantified the ESTIMATE, stromal and immune scores between different groups using the ESTIMATE algorithm.

### 2.4 Differentially expressed genes analysis

Differential analysis was performed using the R package limma. Screening by |log2fold change|>1.2, 289 differential genes were distinguished, with a FDR (false discovery rate) <0.01 between different subtypes derived from principal component analysis (PCA). By unsupervised clustering of 289 genes linked to the distinguishing m5C mode, patients were distinguished into various gene clusters.

### 2.5 Establishment of m5C‐related signature

Because of the complexity and distinguishing m5C mode, we set up a rating system named m5C score to evaluate the distinguishing mode of BLCA patients depend on these signature. Subsequently, PCA was conducted to analyse the 289 genes, scoring two patterns (PC1, PC2) and counting the m5C score for patients individually. The m5C score was calculated like this.

$$m5C\;\text{score}=\sum \left(\mathrm{PC}1i+\mathrm{PC}2i\right)$$

### 2.6 Cell culture, induction and transfection

All cell lines (5637 THP‐1) were purchased from Shanghai institute of cell biology, Chinese academy of sciences and identified by short tandem repeat sequence analysis. All cells were cultured in a humidified environment at 37°C with 5% CO_2_. In the THP‐1 cell line, 185 ng/mL PMA (in DMSO) was added for 6 h, followed by IL‐4 (20 ng/mL) and IL‐13 (20 ng/mL) for 48 h. The THP‐1 cell line was induced into M2 macrophages. The sgRNA of target NSUN6 with pX458 donor was purchased from GenScript. Lipofectamine iMAX (Thermo, USA) was transfected. The cDNA of HDAC10 was synthesized from Invitrogen and cloned into the expression vector pcDNA3.1. Lipofectamine 3000 (Thermo, USA) was transfected according to the directions.

### 2.7 Clinical samples and immunohistochemistry (IHC) and western blots

Twelve cases of tissues who had undergone radical bladder cancer surgery from October to December 2021 were randomly selected from The Affiliated Huai'an Hospital of Xuzhou Medical University and the Second People's Hospital of Huai'an Biobank. Written informed consent was obtained from all patients. According to the immunohistochemical (IHC) results of NSUN6, three samples with the highest expression and three samples with the lowest expression were selected. Immunohistochemistry was performed using the standard immunohistochemistry procedure by Department of Pathology, Jiangsu Cancer Hospital. Anti‐HDAC10 antibody (abcam, #ab108934), anti‐Ki67 antibody (abcam, #ab15580) and anti‐NSUN6 antibody (abcam, #ab214227) were purchased. IHC results were independently evaluated by two senior pathologists. After discarding the media and adding RIPA lysate to the cells, the total protein was extracted. The BCA technique was used to measure the total protein concentration (Thermo). SDS‐PAGE was used to separate the protein samples, and the results were transferred to a PVDF membrane. After blocking with 5% skim milk for 1 h at room temperature, the membrane was washed three times in TBST before being incubated with the primary antibody at the recommended dilution ratio at 4°C overnight. The membrane was then once more washed with TBST before being incubated for 1 h at room temperature with DyLight 680/800‐labelled secondary antibody. The membrane was then given three further TBST washes. Finally, imaging was performed using a fluorescence scanning instrument (Odyssey). Primary antibodies against NSUN6 (17240‐1‐AP), HDAC10 (24913‐1‐AP), GAPDH (60004‐1‐Ig) and HSP90 (60318‐1‐Ig) were bought from Proteintech for this study.

### 2.8 Macrophage chemotaxis assay

M2 macrophages (induced by TPH‐1 cell) was co‐cultured with the 5637 cell line (THP‐1 cells in the upper well and 5637 cells in the lower dish) for 36 h. Cells in the upper part of the well were eluted, and THP‐1 cells in the lower part of the well were stained with CFSE cell dye (ThermoFisher) and photographed using a fluorescence microscope.

### 2.9 m5C RIP‐qPCR

The procedure of m5C immunoprecipitation used methods from the previously reported by Yang et al.^16^, ^17^ In brief, 10 μg of purified mRNAs via TRIZOL method were mixed with 25 μg of anti‐m5C antibody (abcam, #214727) and incubated by rotating at 4°C for 2 h. Then, irradiated three times via Ultraviolet Crosslinker. The mixture was then immunoprecipitated by incubation with Dynabeads Protein A beads at 4°C for 2 h. After mRNA was eluted, it was reverse transcribed into cDNA for qPCR. The primers used were listed in Table S1.

### 2.10 ChIP‐qPCR

Chromatin co‐immunoprecipitation method using Terranova et al. previously reported method.^18^ In brief, after the cell samples were cross‐linked with formaldehyde, they were treated with Covaris M220 Focused‐Ultrasonicator (Peak Power 75, Duty Factor 10.0, Cycles 200, Avg. Power 7.5) at 4–10°C for 10 min, and the DNA fragments were mainly enriched in 200–1000 bp. After sonication, samples were incubated overnight with HDAC10 antibody (abcam, #ab108934) coated protein A magnetic beads. Afterwards, samples were purified and incubated with VAHTS DNA Clean Beads (Vazyme, #N411). Finally, IP samples and input samples were quantified using qPCR. The primers used were listed in Table S1.

### 2.11 Statistical analysis

Spearman correlation analysis was used to calculate the correlation coefficient between m5C regulators expression and TME. We used the Wilcoxon test to calculate the difference between two groups. Kruskal–Wallis test were used for comparison of differences in the case of three or more than three groups. survival curves analysis was analysed using the Tarone–Ware test and Kaplan–Meier (KM) method by R package Survminer v0.49. It is considered statistically significant when *p* < 0.05. All data were processed with R4.0.2 software.

## 3 RESULTS

### 3.1 The m5C regulator's genetic characteristics in BLCA

**FIGURE 1:**
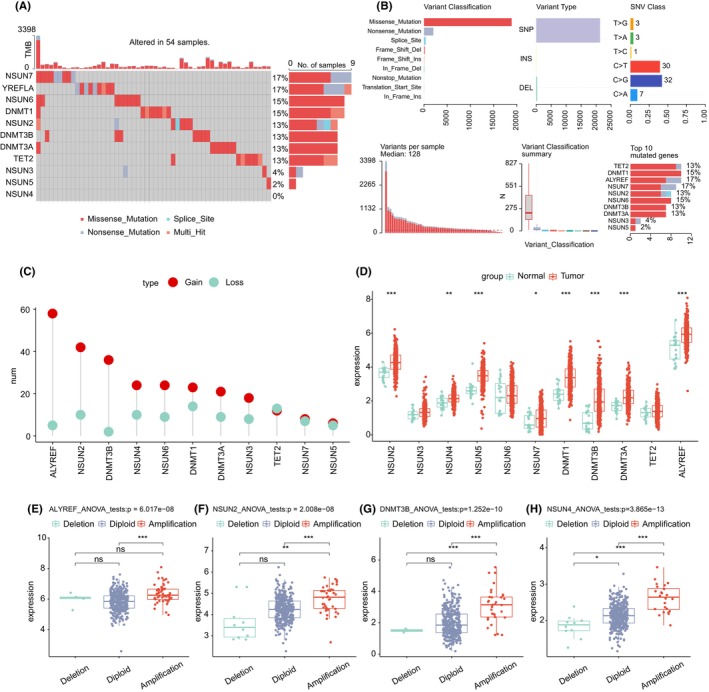
Landscape of m5C regulators in BLCA. (A) Mutation landscape of m5C regulators in BLCA. (B) Mutations of the first 10 genes related to 5‐methylcytosine methylation; (C) The CNV variation frequency of 11 5‐methylcytosine methylation regulators in the BLCA cohort. (D) The expression of 11 5‐methylcytosine methylation regulators in LUAD and lung tissues, tumour, red; normal, green. (E–H) the relationship between CNV and expression of four genes related to 5‐methylcytosine methylation modification. ns, no significant difference; *, *p* < 0.05; **, *p* < 0.01; ***, *p* < 0.001; BLCA, bladder urothelial carcinoma.

This research found 13 m5C‐related regulators, containing nine writers, one reader and one eraser. First, the somatic mutation and CNV of the 11 m5C regulator were summarized in BLCA. Somatic mutations upon the 11 regulators were found in 54 individual patient (Figure1A and Table S1), with an incidence of 13.11% (54/412). The mostly m5C regulators exhibited the similarly incidence of mutation (from 13% to 17%). Three writer, NSUN3, NSUN4 and NSUN5 had almost no mutation (<5%). Figure 1B shows the top 10 genes related to m5C mutations.

### 3.2 CNV analysis in m5C regulators

CNVs, containing amplifications (mean segment >0.3), diploids (−0.3 < mean segment <0.3) and deletions (mean segment<−0.3). The incidences of amplifications and deletions for 11 regulators are performed in Figure 1C. For exploring the connections between gene variants and m5C regulator, the analyses of CNV were conducted, founding that CNV could be a significant element in the disruption of m5C regulator expression.m5C regulator expression was significantly higher in BLCA tissues than in normal tissues (except TET2, NUSN6 and NSUN3) (Figure 1D).

Genes undergoing amplification were connected with high levels of mRNA expression, while genes undergoing deletion were connected with low levels of mRNA expression (Figure 1E–H; Figure S1; Table S2). m5C high incidence of CNV amplification was observed for ALYREF, NSUN2, DNMT3B, NSUN4, DNMT1 and DNMT3A, while a high incidence of CNV deletion was observed for NSUN7 and NSUN5. These gene variants may interrupt m5C signalling transmission in cells, resulting in cellular dysfunction.

Among them, DNMT3b, DNMT1, ALYREF, TET2, NSUN2 and NSUN5 mutations imply that m5C may act abnormally in the tumour. The results of the aforementioned research show that there is a considerable degree of heterogeneity in the hereditary and expression alteration profiles of m5C regulator among BLCA patients, implying that expression change of m5C regulators have a big difference in the tumorigenesis of BLCA.

### 3.3 Eleven regulators mediate m5C methylation modification patterns

By PCA analysis with prCOMP, 11 genes associated with m5C were demonstrated from TCGA (only 11 of the 13 genes connected to m5C alteration have quantifiable expression levels). Figure S2 displays the first three major components determined using pca3d.The samples were distinguished completely between tumour and normal samples. TCGA BLCA patients' mRNA expression told us that mostly m5C regulators are positively related with the other regulators (Figure S2). The 11 m5C regulators were performed to explore the prognostic values(Figure S2) using univariate Cox regression model. And NSUN5 and NSUN6 were proven to be protective factors for BLCA patients.

### 3.4 TME features under distinct m5C modified clusters

**FIGURE 2:**
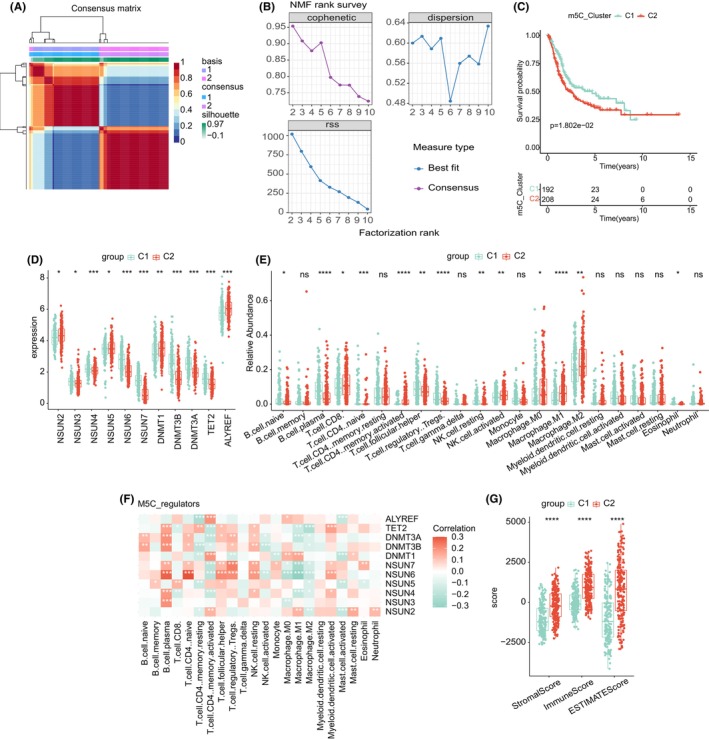
(A) Consensus map of NMF clustering; (B) cophenetic, RSS and dispersion distributions with rank = 2–10; (C) OS survival curves of m5C clusters C1 and C2; (D) expression of 11 genes in two 5‐methylcytosine methylation modification clusters. ns, no significant difference; ***, *p* < 0.001. (E) Relative abundance of 22 immune cells in two 5‐methylcytosine methylation clusters. (F) The correlation between gene expression and relative abundance of 22 immune cells. The score of StromalScore, ImmuneScore and ESTIMATEScore between two 5‐methylcytosine methylation clusters. NMF, non‐negative matrix factorization; OS, overall survival; RSS, residual sum of squares.

Two different modification patterns separated from BLCA patients on the base of 11 m5C genes expression levels by unsupervised clustering (Figure 2A,B). Totally, 400 samples were calculated, of which 192 samples were in C1 and 208 samples were in C2. The patterns were referred as m5C clusters C1 and C2. In addition, we performed prognostic analysis of these two major m5C modification subtypes, and the OS is significantly different between C1 and C2. The patients of m5C cluster C1 has a better survival than C2. Then, the 11 m5C regulators' expression was analysed in the different m5C modification cluster. All of the m5C regulators has a different expression in district cluster, and C1 cluster shows a higher expression of most genes except for NSUN2, NSUN5, DNMT1 and ALYREF (Figure 2D).

### 3.5 TME characteristic of m5C cluster

The immune cells' composition of different m5C modification patterns was analysed by CIBERSORT. Cluster C1 was primarily make up of resting NK cells, B cells plasma, regulatory T cell and follicular T helper cells. While C2 was involved in CD8 T cell, activated CD4 memory T cells, activated NK cell, M0 macrophage, M1 macrophages and M2 macrophages (Figure 2E). The results showed that macrophages were the most infiltrating cells, and C2 was significantly enriched to M0, M1 and M2 macrophages. It indicated that macrophages exert a significant influence in the m5C cluster.

We calculated the relationship between the TME cell infiltration type and m5C‐related signatures using the language's function. As displayed in Figure 2F, the NSUN6 was significantly related to 11 cell, of which, four were consist of immune cells in the C1 clusters (resting NK cells, B cells plasma, regulatory T cell and follicular T helper cells). The remaining five were composed of m5C modified C2 immune cells (activated CD4 memory T cells, activated NK cell, M0 macrophage, M1 macrophages and M2 macrophages). Interestingly, NSUN6 was negatively correlated with the four types of immune cells composed of C1, and was positively correlated with the five immune cells composed of C2. ESTIMATE analysis found that C2 was positively related to the ESTIMATE, immune and matrix scores (Figure 2G).

Among the 11 m5C regulators, we found that NSUN6 was significantly correlated with TME. First, patient with low NSUN6 expression had a poor outcome (Figure S3). Then, we used the ESTIMATE to evaluate BLCA patients, and found that NSUN6 was positively correlated with the matrix, immune and ESTIMATE scores (Figure S3). Moreover, the high levels of NSUN6 expression were found decreased in the immune cells consist of C1 and increased in the immune cells consist of C2 (Figure S3). The connection between the NSUN6 and ICB inhibitors was analysed. We found an association between abnormal NSUN6 and immune dysfunction (Figure S3). Moreover, high expression of NSUN6 group was related to immunity and had a better prognosis.

### 3.6 NSUN6 inhibits the recruitment of M2‐type macrophages

**FIGURE 3:**
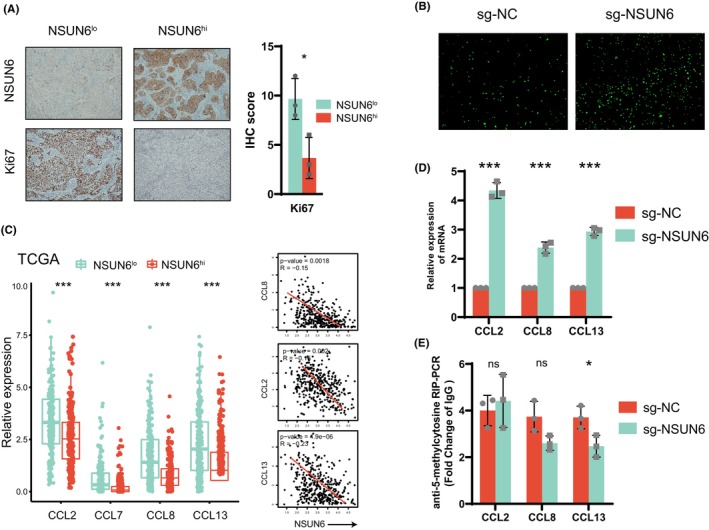
(A) Left: Representative images of immunohistochemical analysis for Ki67 and NSUN6 in six BLCA patients. Right: Quantification of IHC score of each sample. (B) Under the co‐culture condition of M2 macrophages and tumour cells, NSUN6 knockout by sgRNA was used to analyse the changes in the chemotactic ability of tumour cells to chemokines. (C) Correlation of NSUN6 with macrophage‐associated chemokine expression in the TCGA‐BLCA database. (D) After knockout of NSUN6 by sgRNA, the expression of macrophage‐associated chemokines was increased. (E) qRT‐PCR shows, after knockout of NSUN6 by sgRNA, 5‐methylcytosine methylation of the CTCCA motif in the 3′‐UTR region of macrophage‐associated chemokines was not significantly changed.

Above, the connection between low expression of NSUN6, C2 and myeloid‐derived cells were observed, especially macrophages. It has been previously reported that M2 macrophages can promote the malignant progression of tumours by secreting TGFβ, IL6, etc.^11^, ^12^ We hypothesized that NSUN6 inhibits the recruitment of macrophages through some molecular mechanism, resulting in better prognosis in bladder cancer patients. First, we performed NSUN6 immunohistochemistry from 12 bladder cancer samples selected from The Affiliated Huai'an Hospital of Xuzhou Medical University and the Second People's Hospital of Huai'an Biobank, and selected three samples with high NSUN6 expression and three samples with low NSUN6 expression. Anti‐Ki67 immunohistochemistry showed that low NSUN6 expression group had higher malignant potential (Figure 3A).

To further demonstrate the hypothesis that NSUN6 inhibits macrophage recruitment, we constructed NSUN6 knockout 5637 cell line (sgNSUN6) via CRISPR‐Cas9 plasmid (Figure S4,S4). NSUN6 knockout‐cell and NSUN6 wildtype‐cell co‐cultured with the M2‐type macrophages (induced by TPH‐1), respectively. Chemotaxis experiments showed that macrophages were more significantly recruited after knockout of NSUN6 (Figure 3B). This suggests that NSUN6 may negatively regulate chemokine chemotaxis to macrophages. Through TCGA data analysis, macrophage‐related chemokines (CCL2, CCL7, CCL8, CCL13) were significantly increased in NSUN6 low expression samples and C2 (Figure S6). Among them, CCL2, CCL8 and CCL13 were negatively related to the expression of NSUN6 (Figure 3C). We performed PCR validation in the 5637 cell line, which also demonstrated that CCL2, CCL8 and CCL13 mRNA expression was significantly increased after NSUN6 knockout (Figure 3D).

Previous literature reports that NSUN6, as an m5C methylation writer, often promotes mRNA expression by mediating hypermethylation, which is obviously inconsistent with the above trend. Therefore, we examined the m5C methylation levels at the methylation modification sites in the 3′ UTR regions of CCL2, CCL8 and CCL13. The results displayed that the m5C methylation levels of CCL2, CCL8 and CCL13 were not significantly or slightly decreased after NSUN6 knockout (Figure 3E). In addition, we performed cell function experiments for NSUN6 KO cells and WT cells. The results showed that, the malignant phenotypes were significantly inhibited by NSUN6 knockout (Figure S4). We speculate NSUN6 can inhibit the malignant progression of lung adenocarcinoma in two ways: (1) Inhibiting the malignant phenotypes of tumour cells through other pathways; (2) Inhibiting the malignant progression of lung adenocarcinoma by inhibiting macrophages from interfering with the immunosuppressive microenvironment. In conclusion, we found that the m5C methyltransferase NSUN6 inhibited the recruitment of macrophages, possibly not by directly regulating the expression of m5C methylation of CCL2, CCL8 and CCL13.

### 3.7 NSUN6 suppresses macrophage‐associated chemokine expression by regulating the histone deacetylase family

**FIGURE 4:**
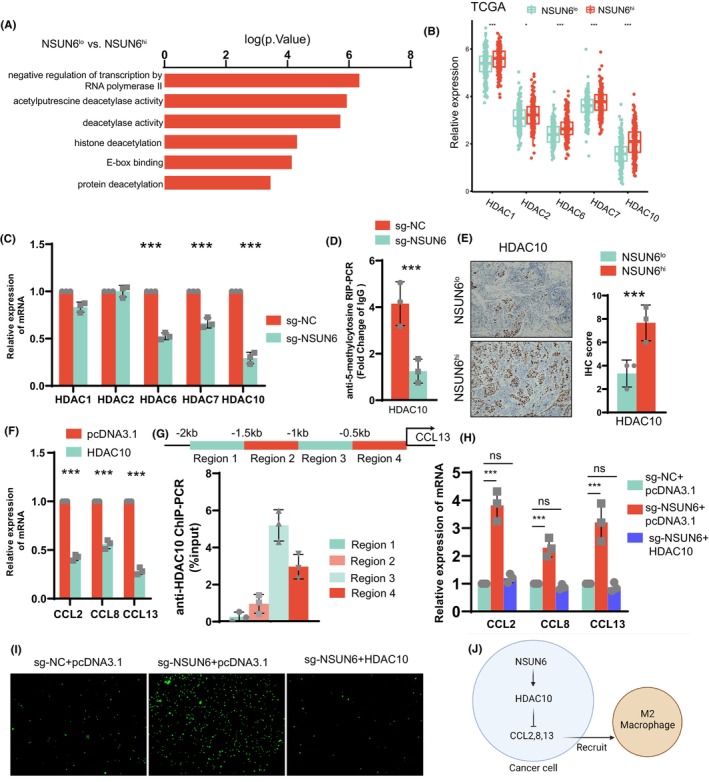
(A) Patients in the TCGA‐BLCA dataset were divided into two groups according to the expression level of NSUN6 and subjected to differential gene expression analysis, and the results were subjected to GO pathway enrichment analysis. (B) Patients in the TCGA‐BLCA dataset were divided into two groups according to NSUN6 expression levels, and differences in HDAC family mRNA expression were analysed. (C) By PCR, it was found that the mRNA expression of HDAC family was significantly increased before and after sgRNA knockout of NSUN6. (D) qRT‐PCR shows, after knockout of NSUN6 by sgRNA, 5‐methylcytosine methylation of the CTCCA motif in the 3′‐UTR region of HDAC10 was upregulated significantly. (E) Immunohistochemical analysis of HDAC10 in 6 BLCA patients. (F) qRT‐PCR detection shows that the expression of macrophage‐related chemokines increased after overexpression of HDAC10. (G) ChIP‐PCR showed that HDAC10 was significantly bound to chromatin in a region 0.5–1 kb upstream of the CCL13 transcription start site. (H) qRT‐PCR showed that the downregulation of macrophage‐associated chemokine expression by sgRNA knockout of NSUN6 was reversed by HDAC10. (I) Co‐culture chemotaxis experiments of macrophages and tumour cells showed that the reduction of macrophage chemotaxis by sgRNA knockout of NSUN6 was reversed by HDAC10. (J) Mechanism of higher macrophage recruitment in BLCA patients with high NSUN6 expression.

**FIGURE 5:**
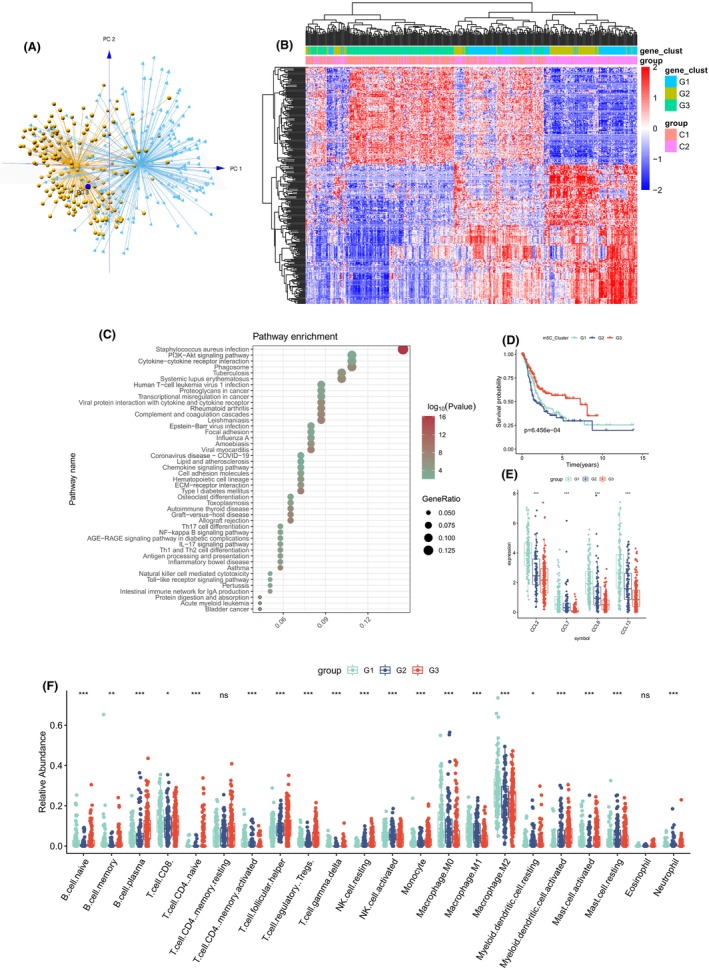
(A) PCA for two 5‐methylcytosine methylation modification patterns to distinguish 5‐methylcytosine methylation clusters C1 and C2. (B) Unsupervised clustering of overlapping 5‐methylcytosine methylation phenotype‐related genes was performed to classify patients into different genomic subtypes, termed 5‐methylcytosine methylation gene clusters G1–G3. (C) KEGG enrichment analysis of 289 5‐methylcytosine methylation phenotype‐related genes. (D) OS survival curves of 5‐methylcytosine methylation gene clusters G1, G2 and G3. (E) Expression of macrophage‐related chemokines in 5‐methylcytosine methylation gene clusters. (E) Relative abundance of 22 immune cells in three 5‐methylcytosine methylation gene clusters.

We performed differential expression analysis of the transcriptome expression profiles in patients with low and high expression of NSUN6 from TCGA‐BLCA. Pathway enrichment analysis showed that multiple pathways of histone deacetylation and negative transcriptional regulation are enriched (Figure 4A). Therefore, we compared the expression of histone deacetylase family mRNAs in the two groups of patients. As expected, HDAC1, HDAC2, HDAC6, HDAC7 and HDAC10 were all up‐regulated in the NSUN6 high expression group and C1 group (Figure 4B,C). We observed the same trend in the 5637 cell line (Figure 4C). We selected HDAC10 with the most significant difference for follow‐up research. After NSUN6 knockout, the m5C methylation level of the methylation modification site in HDAC10 3′UTR region was significantly decreased (Figure 4D). This suggests that HDAC 10 is elevated through NSUN6‐mediated m5C methylation. Immunohistochemistry of bladder cancer samples also demonstrated that HDAC10 expression was also significantly elevated in NSUN6‐high samples (Figure 5E).

Next, we further explored whether HDAC10 could directly regulate the expression of macrophage‐associated chemokines. We overexpressed HDAC10 in the 5637 cell line (Figure S4, S4), and the mRNA expressions of CCL2, CCL8 and CCL13 were significantly decreased (Figure 4F). In order to verify the transcriptional regulation ability of HDAC10 for chemokines, we designed an anti‐HDAC10 ChIP‐PCR for 2 kb upstream of the transcription start site (promoter region), using CCL13 as an example. The results showed that HDAC10 was significantly bound within 1 kb downstream of the CCL13 transcription start site, suggesting a high level of deacetylation (Figure 4G). Finally, we knocked down NSUN6 in the 5637 cell line, and the expression of CCL2, CCL8 and CCL13 was significantly increased. Meanwhile, after overexpression of HDAC10, this trend was reversed (Figure 4H). Similarly, macrophage chemotaxis assays also demonstrated increased recruitment of M2 macrophages by tumour cells after knockout of NSUN6. This trend was reversed after overexpression of HDAC10 (Figure 4I). In addition, HDAC10 overexpression seem did not alter the major malignant phenotypes of lung adenocarcinoma cell lines, including proliferation and migration (Figure S4). In conclusion, we demonstrated that NSUN6 promoted HDAC10 expression by mediating m5C methylation, inhibited the transcription of macrophage‐associated chemokines and thus inhibited the recruitment of M2‐type cells (Figure 4J). This may partly explain the mechanism of better prognosis in patients in C2 or high expression of NSUN6.

### 3.8 Generation and functional enrichment analysis of m5C signatures

289 differentially expressed genes (DEGs) correlated with the m5C cluster was selected by using R package Limma. PCA analysis showed that they were separated from C1 and C2 group (Figure 5A). Then, we divided patients into three subtypes by unsupervised clustering of 289 m5C‐related DEGs (Figure S6). We named these three subtypes m5C gene subtype G1–G3. It was discovery that most patients in gene subtype G1 and G2 coincided in cluster C2 and the most patients of Gene subtype G3 were also included in cluster C1.

To further discovery the key biological processes, which these 289 DEGs may involve in, we used R package cluster Profiler v4.0.5 for KEGG enrichment analysis and showed top 30 pathways(*q* < 0.05): Transcriptional dysregulation in cancer, bladder cancer, chemokine signalling pathway, cellular sense, cell adhesion molecules, ECM−receptor interaction, PI3K − Akt signalling pathway, NF‐kappa B signalling pathway, IL − 17 signalling, Th17 cell differentiation (Figure 5C). The 289 DEGs were related to m5C modification and were significantly correlated with TME and tumorigenesis. Then, the relative infiltration abundance of the three subtypes of macrophages decreased from G1 to G2 to G3. Thus, we had found that the critical role of m5C modification in TME, as well as the 289 DEGs. And macrophages are the most affected. Moreover, KM curve was analysed among gene subtypes G1–G3, and found a close connection between subtype and prognosis (*p* < 0.001, Figure 5D).

The prognosis of G3 was superior to that of G1 and G2. Significantly, G3 had the lowest macrophage‐associated chemokines expression and highest histone deacetylation associated gene expression among m5C‐related gene subtype (Figure 5E); Figure S5.

### 3.9 Construction of m5C score model

Because of the complexity and heterogeneity, a m5C score model was built to evaluate the individual BLCA patients' m5C pattern. First, we selected 92 genes correlated with prognosis (*p* < 0.05) from 289 DEGs. We exhibited the univariate COX analysis result of the 92 genes in Table S2. Then PCA analysis were carried out and the m5C‐score were calculated: m5C‐score = ∑PC1i + PC2i. Table S3 exhibited the exactly result of each BLCA patients.

**FIGURE 6:**
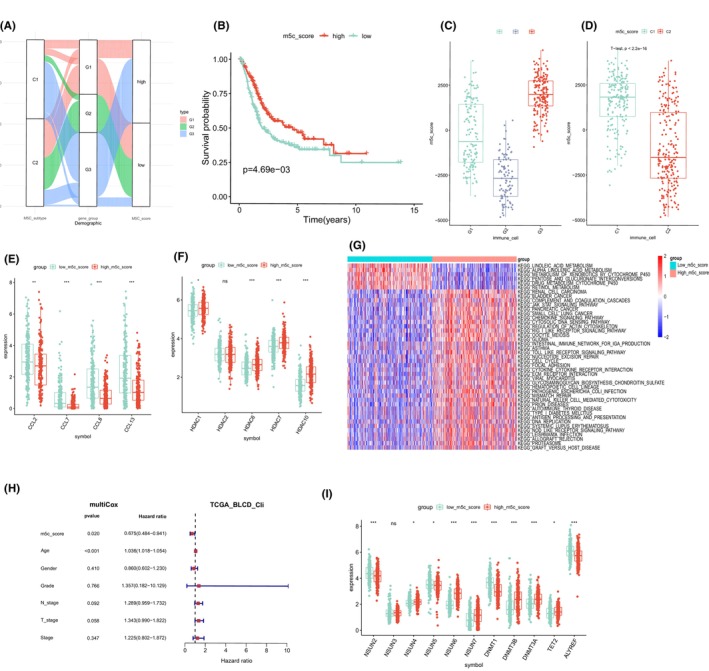
(A) Alluvial diagram showing the changes in 5‐methylcytosine methylation clusters, gene clusters and 5‐methylcytosine methylation scores. (B) Survival analyses for both low and high 5‐methylcytosine methylation score patient group. (C) Distribution of patients with different 5‐methylcytosine methylation scores in two 5‐methylcytosine methylation modification clusters. (D) Distribution of patients with different 5‐methylcytosine methylation scores in three 5‐methylcytosine methylation gene clusters. (E) Expression of macrophage‐related chemokines in low and high 5‐methylcytosine methylation score patient group. (F) Expression of histone deacetylase family mRNAs in both low and high 5‐methylcytosine methylation score patient groups. (G) GSVA enrichment analysis showing the activation states of biological pathways in different 5‐methylcytosine methylation score groups. (H) Multivariate Cox regression analysis for 5‐methylcytosine methylation score in BLCA patients shown by forest plot. (I) Expression of 11 5‐methylcytosine methylation regulators in both low and high 5‐methylcytosine methylation score patient groups.

The patients were selected to high and low m5C score group on the base of median of m5C score, and alluvial diagram demonstrated that the difference among m5C clusters, gene subtype and m5C score (Figure 6A). The result showed that m5C cluster C1 and gene subtype G3 subtype had better prognosis and higher m5C score.

In order to investigate the relationship between BLCA patients' prognosis and m5C score, we divided patients into high and low m5C score group depended on the median score. We then performed survival analysis between the two group, and the result showed a better prognosis of the high m5C score patients, which was in line with the result above (Figures 2C and 5D). m5C significant difference of the m5C score among subtypes is shown in Figure 6C, it was also confirmed that higher score was connected with a better outcome, with cluster G3 ranking the highest and cluster G2 ranking the lowest. Moreover, there was a big difference between cluster C1 and C2 (Figure 6D). The m5C score of cluster C1 was apparently higher than cluster C2, and cluster C1 showed a better prognosis, which proved that a higher m5C score was related to a better prognosis. Thus, high m5C scores were associated with better outcome and low m5C scores were associated with worse outcome in BLCA patients. As same as the result above, the patient with high m5C score had low macrophage‐associated chemokines expression and high histone deacetylation associated gene expression than patient with low m5C score (Figure 6E,F).

GSVA analysis was performed to discovery the key pathway between the different m5C score group. The result displayed that the pathway related to the high score patients was primarily related to DNA repair, stroma and cell cycle, while patients with low score was linked with linoleic acid metabolism, metabolism of xenobiotics by cytochrome P450 and alpha linolenic acid metabolism (Figure 6G).

Moreover, multivariate Cox regression analysis told us that m5C score was an independent prognostic factor (Figure 6H). Furthermore, 11 m5C regulators were analysed between the different m5C score group. Seven regulators' expression was corresponded to m5C score. Figure 6I exhibited that a lower regulators expression (NSUN2, NSUN5, DNMT1, ALYREF) was associated with a higher m5C score excluded NSUN6, DNMT3B, DNMT3A, TET2.

## 4 DISCUSSION

Accumulating evidence demonstrated that abnormal of epigenetic modifications was involved in progression and methylation regulators was detected to predict the survival and diagnosis of cancer.^19^ m5C is the one of most preventative DNA modification in eukaryotes while emerging evidence has uncovered that RNA m5C and its regulators exerted significant roles in posttranscriptional regulation. For example, ALYREF and NSUN4 were identified to be promising targets for HCC treatment.^20^ In addition, aberrant NSUN2 mediated m5C‐modified H19 lncRNA, which facilitated the tumorigenesis of HCC by engaging the oncoprotein G3BP1.^21^ Increasing evidence showed that m5C modifications could adjust the tumour microenvironment (TME) and affect the behaviours of immune cells.^21^, ^22^ Comprehensive understanding of m5C modifications and cross‐talk between m5C and TME draws widely attention, recently. Chen et al. identified two m5C methylation modification mode with remarkably distinct TME immune cell infiltration characterisation, which predicted the outcome of lung adenocarcinoma (LUAD).^6^ In addition, Zhang et al. constructed a novel m5C‐related lncRNAs signature (m5CrLS scores) to discriminate different immunosuppressed state, which would guide the efficacy of immunotherapy.^23^ As most conclusions have uncovered the comprehensive analysis relationship between m5C and TME and identified distinct phenotypes, there remains a gap in recognition of how m5C modifications adjust TME and regulate the behaviour of immune cells.

In this work, we first analyse the genetic mutations and CNV of 11 m5C regulators in BLCA samples, which performed expressional alteration landscape and genetic characteristics in m5C regulators, which indicated a significant correlation between m5C regulators and tumorigenesis of BLCA. Also, we demonstrated two distinct m5C methylation modification patterns (C1&C2) with different TME cell infiltration characteristics based on the expression quantity of 11 m5C regulators. C1 was primarily make up of resting NK cells, follicular T helper cells, regulatory T cell and plasma B cells, while C2 was involved in CD8 T cell, activated NK cells, activated CD4 memory T cell, M0 Macrophage, M1 macrophages and M2 macrophages. Among them, we found an interest result that NSUN6 was associated with the types of immune cells, especially M2 macrophages. Individual analysis displayed again that high NSUN6 gene expression group was correlated with immune function of TME and had a better prognosis. Further experiment identified that NSUN6 could inhibit the recruitment of M2 macrophages which promoted the progression of BLCA via regulating the expression of m5C methylation of CCL2, CCL8 and CCL13 in the 3'UTR regions. Mechanically, we revealed that NSUN6 could promote the expression of HDAC10 by mediating m5C methylation, then inhibited the transcription of macrophage‐associated chemokines (CCL2 and others), and thus hindered the recruitment of M2 macrophages. HDAC10 acts as a histone deacetylase to mediate global histone deacetylation and repress target gene transcriptional activity. However, due to the lack of public ChIP‐Seq data of HADC10 in lung cancer tissues/cells, our regulatory targets of HDAC10 have not been confirmed. Our experimental results demonstrated that HDAC10 was significantly enriched at the promoter of CCL13, compared to IgG. Therefore, we believe that HDAC10 inhibits the transcription of CCL13 by binding to the CCL13 promoter. In addition, a scoring system was proposed to evaluate the prognosis of BLCA, which found that patients with a better prognosis predominantly displayed a high m5C score, and the majority of the C1 sample were identified with G3 sample with better outcome. Also, the patient with high m5C score had low macrophage‐associated chemokines expression and high histone deacetylation associated gene expression than patient with low m5C score. In order to further determine the biological processes involving in the m5C patterns, functional enrichment analyses screened a total of top 30 pathways including transcriptional dysregulation in cancer, bladder cancer, chemokine signalling pathway, cellular sense, cell adhesion molecules, ECM−receptor interaction, NF‐kappa B signalling pathway, Th17 cell differentiation, PI3K‐Akt signalling pathway and IL‐17 signalling. Above results provide novel insight for clinical application of m5C methylation modifications in BLCA and partly elucidate the regulation mechanism of m5C in TME reprogramming, which would aid to survival analysis and discovery of new immunotherapy or treatment strategies. In previous studies, m5C methylation at both RNA and DNA levels was associated with the malignant progression of bladder cancer. However, due to clinical sample collection and preservation, researchers can hardly classify bladder cancer at the molecular level simply based on m5C methylation levels. Therefore, we established m5C‐cluster in the TCGA‐BLCA dataset using the expression of 5mC methylation regulatory factors that have been widely reported previously. This method has many advantages: (1) Expression levels at both RNA and protein levels can be obtained by qPCR, IHC, etc. (2) Its wider range of applications is more convenient for subsequent queue verification. However, the disadvantages of this method are also obvious. For the m5C‐cluster classification, it is difficult for us to fully explain whether the differentially expressed genes downstream of each cluster are regulated by abnormal m5C methylation levels, or a more complex regulatory network and cascade reaction. Therefore, based on the potential downstream genes of m5C‐cluster, we constructed m5C‐cluster‐related gene‐clusters using unsupervised clustering. The m5C‐cluster‐related gene‐clusters have good resolution, classification ability and potential vulnerabilities.

Recent evidence revealed an obvious correlation between m5C modulators and immune infiltration.^24^, ^25^ The methylation modification patterns carry out an important function in the TME, such as promoting M2‐type macrophage polarisation, downregulating tumour‐associated antigens and inhibiting CTL function. Chen et al. identified two distinct m5C modification patterns discriminating the TME diversity and complexity and proposed a m5C based score system to post implications for tumour immunotherapy, predicting prognosis of LUAD patients.^6^ Li et al. established a m5C‐lncRNA based nomogram, which predicted the prognosis and showed negative correlation with TMB.^25^ Besides, several other studies developed a m5C‐based risk model to evaluate the infiltration characterisation in TME and estimate the survival time, which showed perspective value on understanding of m5C methylation modification in TME.^19^, ^24^ Thus, we performed our analysis in BLCA and provoked significant correlation between m5C and TME. Different from previous studies, we found an interest phenotype that low levels of NSUN6 some was found increased in the immune cells composed of C1, and significantly decreased in the immune cells composed of C2 and was significantly related to the different types of immune cells, especially M2 macrophages. Increasing evidence modulated that m6A methylation mediated the behaviour of immune cells such as macrophages.^26^, ^27^ Yin et al. elaborated that METTL3 impairs the YTHDF1‐mediated translation of SPRED2, activating NF‐kB and STAT3 through the ERK pathway, which increased M1/M2‐like tumour‐associated macrophage.^26^ Thus, we further explored how m5C modification modulate the recruitment of M2 macrophages. Bioinformatics and experimental analysis told us that NSUN6 could promote the expression of HDAC10 by mediating m5C methylation, inhibiting the transcription of macrophage‐associated chemokines (CCL2 and others), and thus hindered the recruitment of M2 macrophages. These findings partly mechanically illustrate the importance of m5C modification mode in modulating the TME reprogramming, which provided novel insight in understanding the correlation between m5C methylation modifications with TME.

There are still some limitations in our study. Our score system did not include many clinic pathological parameters, which might hinder the precise of prediction. In addition, our study proposed the hypothesis of NSUN6 modulating the macrophages. Further experiment should be taken to sustain the result. Furthermore, we did not collect the information from BLAC patients receiving the ICBs, which might be more valuable for predicting the response rate of immunotherapy.

To sum up, the present study provided the landscape of m5C modification patterns inducing TME complexity and heterogeneity and illustrated the modulating mechanism of m5C‐mediating NSUN6 in the recruitment of macrophages, which shed more light on the relationship between m5C methylation modifications with TME cell‐infiltrating features.

## CONSENT STATEMENT

Written informed consent was obtained from all patients.

## AUTHOR CONTRIBUTIONS

**Dali Yan:** Investigation (equal); writing – original draft (equal). **Yongsong Xie:** Formal analysis (equal); software (equal). **Liyuan Huang:** Methodology (equal); resources (equal); software (equal). **Yi zhang:** Formal analysis (equal); visualization (equal); writing – review and editing (equal). **Runhuan Gu:** Data curation (equal); resources (equal); validation (equal). **huaibing xie:** Conceptualization (equal); validation (equal); visualization (equal). **xing huang:** Conceptualization (equal); supervision (equal); validation (equal). **hao luo:** Conceptualization (lead); supervision (equal).

## CONFLICT OF INTEREST STATEMENT

The authors declare no conflicts of interest in this work.

## Supporting information


Figure S1.

Figure S2.

Figure S3.

Figure S4.

Figure S5.

Figure S6.

Table S1.

Table S2.

## Data Availability

Data available on request due to privacy/ethical restrictions
