# Supplementary material for: RNA m5C methylation orchestrates BLCA progression via macrophage reprogramming
Source: J Cell Mol Med. 2023 Jul 5;27(16):2398–411. doi: 10.1111/jcmm.17826 (PMC10424284; doi:10.1111/jcmm.17826)
Supplement: Supplementary file 1 — Figure S1. [file JCMM-27-2398-s001.pdf]

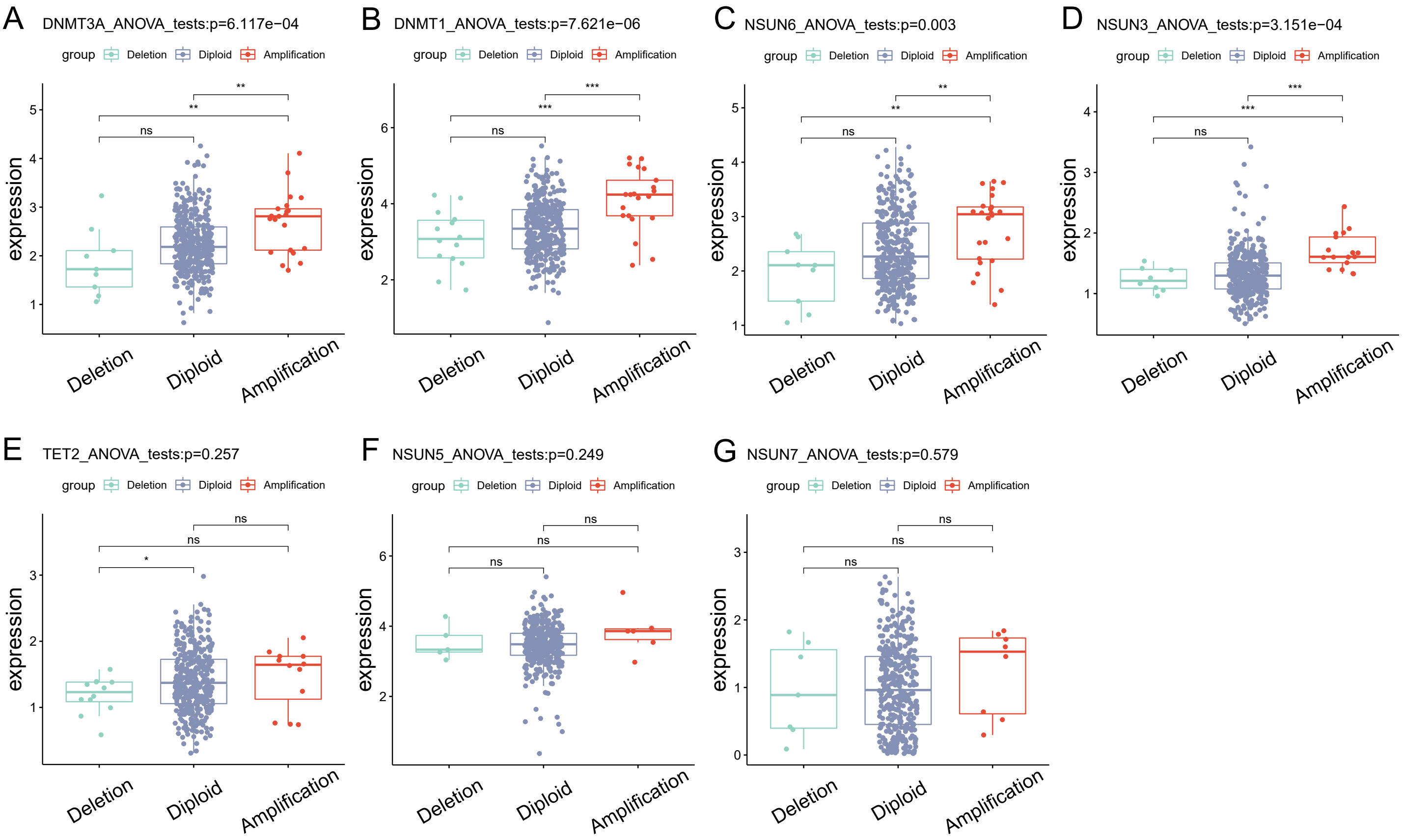
A
DNMT3A_ANOVA_tests:p=6.117e−04
B
DNMT1_ANOVA_tests:p=7.621e−06
C
NSUN6_ANOVA_tests:p=0.003
D
NSUN3_ANOVA_tests:p=3.151e−04
E
TET2_ANOVA_tests:p=0.257
F
NSUN5_ANOVA_tests:p=0.249
G
NSUN7_ANOVA_tests:p=0.579
group
Deletion
Diploid
Amplification
expression
ns
*
**
***
