# Supplementary figures and images for: RNA m5C methylation orchestrates BLCA progression via macrophage reprogramming
Source: J Cell Mol Med. 2023 Jul 5;27(16):2398–411. doi: 10.1111/jcmm.17826 (PMC10424284; doi:10.1111/jcmm.17826)

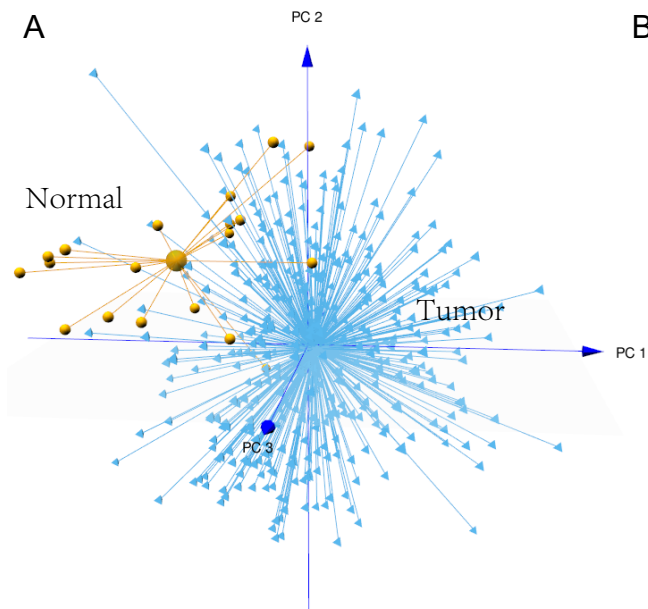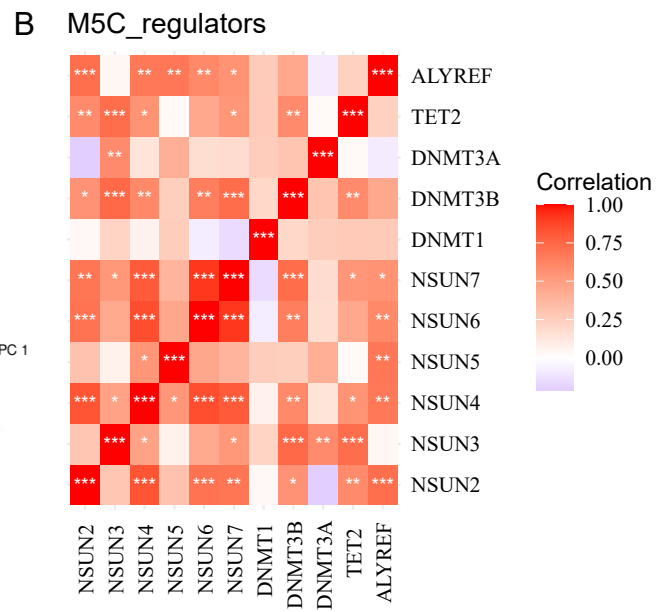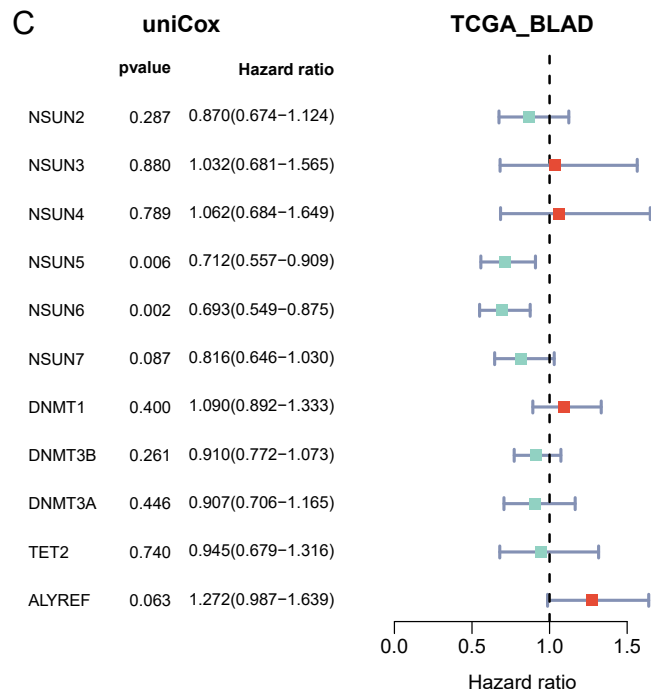

Supplement: Supplementary file 2 — Figure S2. [file JCMM-27-2398-s008.pdf]

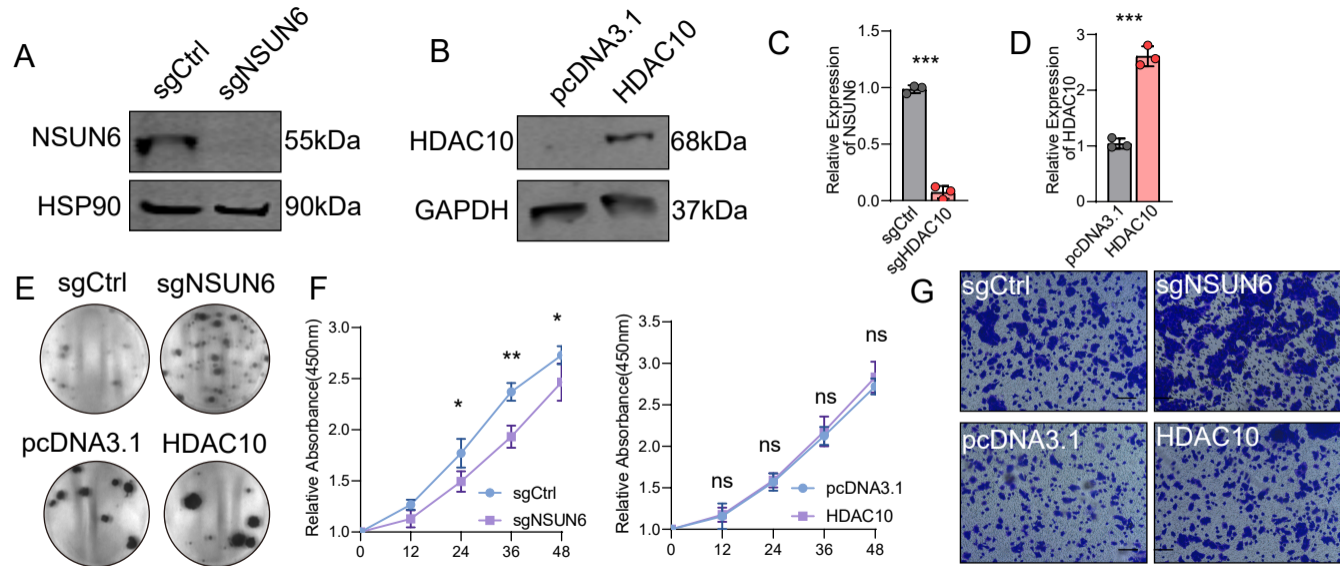

Supplement: Supplementary file 4 — Figure S4. [file JCMM-27-2398-s007.pdf]

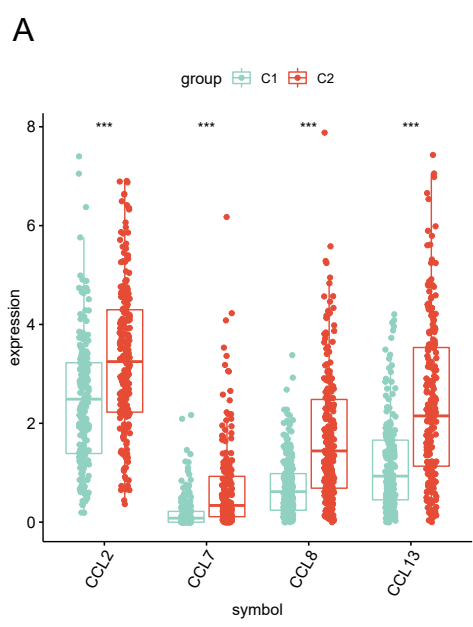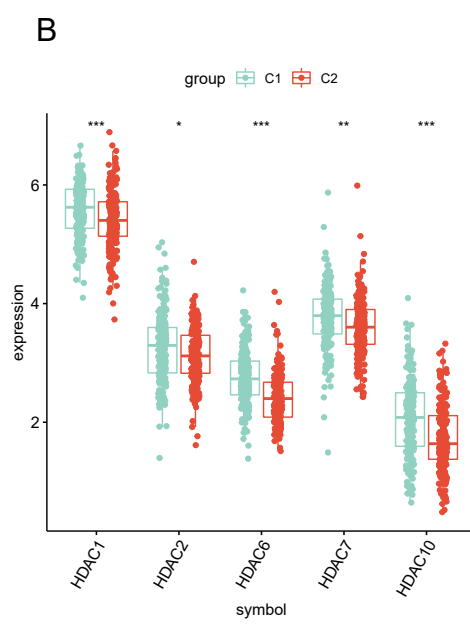

Supplement: Supplementary file 5 — Figure S5. [file JCMM-27-2398-s002.pdf]

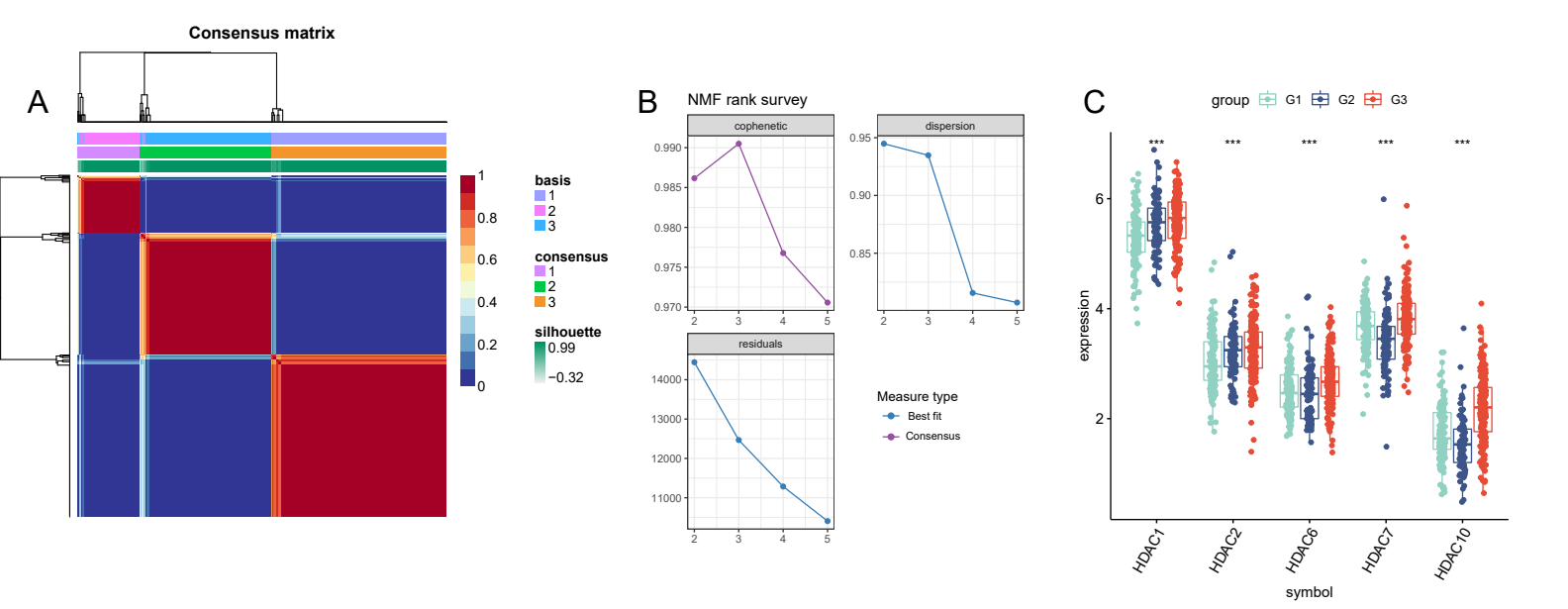

Supplement: Supplementary file 6 — Figure S6. [file JCMM-27-2398-s003.pdf]
