# Supplementary material for: RNA m5C methylation orchestrates BLCA progression via macrophage reprogramming
Source: J Cell Mol Med. 2023 Jul 5;27(16):2398–411. doi: 10.1111/jcmm.17826 (PMC10424284; doi:10.1111/jcmm.17826)
Supplement: Supplementary file 7 — Table S1. [file JCMM-27-2398-s006.docx]

Table S1. The primers sequence

| Names | Oligonucleotide sequence |
| --- | --- |
| R1-F | tggaatgtgtgtctgaaggga |
| R1-R | tcctccaagcactgaaggaa |
| R2-F | cagtggtgcaatcttggctc |
| R2-R | agttctagagcagcctggac |
| R3-F | ggatgagtgggaaagggaca |
| R3-R | tagagctgttgatgggaggg |
| R4-F | cagaaggaagccatgccatg |
| R4-R | gggagtggagaagcttggaa |
| CCL2 3'UTR -F | acactcactccacaacccaa |
| CCL2 3'UTR -R | gcaatttccccaagtctctgt |
| CCL8 3'UTR -F | ccaggtgcagtgtgacatta |
| CCL8 3'UTR -R | acagacaggtaggagggaga |
| CCL13 3'UTR -F | tcctctggcctcctcttcta |
| CCL13 3'UTR -R | ccgaatacaaacccactgcc |
| HDAC10 3'UTR -F | tacaccgcagaaatgacacc |
| HDAC10 3'UTR -R | gaggtgaggtgaggggtg |

Table S3. m5C score for TCGA patients.

|  | PC1 | PC2 | m5C score |
| --- | --- | --- | --- |
| TCGA-FD-A3SS-01A | -3.371152545 | -0.702349049 | -1194.156979 |
| TCGA-FD-A43S-01A | 0.89542906 | 3.700815107 | 1793.683222 |
| TCGA-UY-A78K-01A | 4.89248044 | 2.306307182 | 2490.26828 |
| TCGA-DK-A2HX-01A | -2.825186347 | 5.104507252 | 753.1872744 |
| TCGA-DK-A1A3-01A | -0.286497131 | 2.180577856 | 540.8206375 |
| TCGA-GC-A6I3-01A | 3.915138581 | 3.079398569 | 2549.284475 |
| TCGA-XF-AAMJ-01A | -1.180490094 | 7.647439196 | 2564.822377 |
| TCGA-GC-A6I1-01A | -9.986374838 | -3.338797293 | -3650.361448 |
| TCGA-ZF-A9RL-01A | 11.12154517 | -3.223716766 | 2525.638711 |
| TCGA-DK-A3IL-01A | 4.103115797 | 2.854867891 | 2503.144879 |
| TCGA-E7-A6MF-01A | 4.491182885 | -4.55745591 | -17.1306996 |
| TCGA-BT-A20R-01A | -3.307763195 | 4.808004813 | 476.6437514 |
| TCGA-H4-A2HQ-01A | 3.636670241 | -3.885308814 | -68.26265792 |
| TCGA-GV-A3QH-01A | 9.741096899 | -2.643328809 | 2216.340246 |
| TCGA-4Z-AA82-01A | -8.727290107 | 0.00521134 | -2777.335733 |
| TCGA-E7-A4XJ-01A | 6.090228679 | -0.570805492 | 1963.088952 |
| TCGA-XF-A8HE-01A | -8.004162899 | -4.279794456 | -3666.72851 |
| TCGA-XF-A9T0-01A | 3.157524875 | 3.496583944 | 2485.804842 |
| TCGA-FD-A5BT-01A | -9.780766304 | 3.296499233 | -1791.925576 |
| TCGA-FD-A3SN-01A | -4.644062973 | 1.902398297 | -899.9056328 |
| TCGA-G2-AA3C-01A | -9.653221803 | 0.190646896 | -2039.946349 |
| TCGA-HQ-A2OE-01A | 6.264116509 | -2.9868577 | 880.0166689 |
| TCGA-BL-A3JM-01A | -4.165345139 | -1.961461136 | -1346.624139 |
| TCGA-XF-AAN3-01A | -3.2983378 | 0.904306246 | -767.4465714 |
| TCGA-FD-A3SR-01A | -1.042060265 | 3.169515902 | 643.2610588 |
| TCGA-4Z-AA89-01A | 9.892633144 | -4.104488501 | 1870.884613 |
| TCGA-G2-A2EO-01A | 0.364587412 | 3.482579261 | 1438.449033 |
| TCGA-ZF-AA5H-01A | -8.278687924 | 1.29471125 | -2011.095608 |
| TCGA-BT-A20V-01A | -2.998555883 | -3.444422671 | -1629.607459 |
| TCGA-GC-A3WC-01A | -6.112197565 | -3.985640227 | -2654.101657 |
| TCGA-GV-A3JV-01A | 4.987237502 | 2.85102191 | 2717.461606 |
| TCGA-YF-AA3M-01A | 0.059018276 | -1.575971873 | -481.0554716 |
| TCGA-FD-A3B5-01A | -9.178361772 | -2.667244393 | -3931.355874 |
| TCGA-CF-A47V-01A | 8.856175514 | 0.054571305 | 3072.726925 |
| TCGA-GU-A42P-01A | 6.337845501 | -0.660875945 | 1800.503433 |
| TCGA-BT-A20T-01A | 0.375883581 | 2.130028258 | 917.5373897 |
| TCGA-XF-AAN0-01A | 0.38085484 | -1.061493732 | -200.5096402 |
| TCGA-E7-A7DV-01A | -6.918984079 | 2.03773645 | -1365.352808 |
| TCGA-GV-A40E-01A | -6.555055917 | 0.536367137 | -1880.862298 |
| TCGA-FJ-A3Z9-01A | 6.855554109 | -2.365724756 | 1571.763637 |
| TCGA-XF-A9ST-01A | -2.559328256 | -3.136191169 | -1136.973433 |
| TCGA-FJ-A3ZF-01A | 10.18265896 | -0.414057114 | 3193.270111 |
| TCGA-YC-A89H-01A | -0.070209733 | 1.48474759 | 541.4326194 |
| TCGA-CF-A9FM-01A | 8.739227629 | -2.065956594 | 2137.898284 |
| TCGA-DK-A1AD-01A | 5.265115619 | 2.61112757 | 2918.415637 |
| TCGA-G2-A2EK-01A | 6.727541089 | 0.993222137 | 2748.291155 |
| TCGA-E5-A2PC-01A | 0.144916401 | -4.220372611 | -1053.802261 |
| TCGA-C4-A0F1-01A | -10.85293684 | -4.68940016 | -4125.708837 |
| TCGA-FD-A43N-01A | 3.212214565 | 3.845758864 | 2759.384381 |
| TCGA-BL-A13J-01A | -2.292280984 | 1.791314735 | -179.5711166 |
| TCGA-FD-A3SO-01A | -8.521707295 | -0.426580808 | -2665.110475 |
| TCGA-XF-A8HD-01A | -1.789665551 | 2.035877652 | 73.17116156 |
| TCGA-BT-A20X-01A | -10.85408154 | -4.271930418 | -4273.071945 |
| TCGA-ZF-AA52-01A | -2.828947828 | 5.108793276 | 780.3445906 |
| TCGA-DK-AA76-01A | 9.023576252 | -2.89752461 | 1926.788925 |
| TCGA-GV-A3JW-01A | 9.272509373 | -0.388099553 | 3349.184824 |
| TCGA-FD-A3N6-01A | -10.18122689 | -7.589850115 | -4821.389003 |
| TCGA-CU-A5W6-01A | 5.419404937 | 0.553226714 | 2101.690673 |
| TCGA-DK-A2I4-01A | -4.668502872 | 2.990590288 | -500.3536809 |
| TCGA-DK-AA6M-01A | -7.54827457 | 0.295540317 | -2068.797381 |
| TCGA-4Z-AA7Y-01A | 5.59707749 | -2.788726172 | 868.2225983 |
| TCGA-DK-A3WY-01A | -6.956919496 | 0.589199835 | -1498.14648 |
| TCGA-E7-A3X6-01A | -0.919746466 | -2.089177956 | -940.2812759 |
| TCGA-K4-A3WV-01A | -3.542951722 | -3.306841097 | -2040.203845 |
| TCGA-DK-A3IQ-01A | -7.525826787 | 6.389618674 | -362.2928347 |
| TCGA-ZF-AA56-01A | -2.927655326 | -0.670209961 | -1304.144887 |
| TCGA-CU-A3QU-01A | 10.77703837 | -1.551828407 | 2978.324223 |
| TCGA-FD-A6TI-01A | 1.897095036 | 1.052640255 | 1032.796391 |
| TCGA-XF-A9T3-01A | -7.2295783 | 3.348588933 | -1250.118999 |
| TCGA-FD-A3B3-01A | -11.85298937 | -0.94002476 | -4144.078856 |
| TCGA-YF-AA3L-01A | 7.817773827 | -0.118885927 | 2502.212233 |
| TCGA-SY-A9G0-01A | -1.743757386 | 5.13902744 | 1211.047096 |
| TCGA-ZF-A9R2-01A | 5.855838066 | -5.070701105 | 222.5440568 |
| TCGA-GU-AATQ-01A | -4.259314237 | -2.783927637 | -2004.760794 |
| TCGA-FD-A5C1-01A | -8.781129355 | 0.516144483 | -2611.094872 |
| TCGA-DK-A2I1-01A | 3.251329252 | 6.200444688 | 3858.224708 |
| TCGA-CF-A5U8-01A | 10.42488275 | -2.394143164 | 2634.810813 |
| TCGA-UY-A9PB-01A | -10.39675178 | 2.387538701 | -2406.651837 |
| TCGA-DK-A3X1-01A | 3.70089395 | 1.790146958 | 1860.033424 |
| TCGA-DK-A1AC-01A | 4.18789782 | 1.76764047 | 1880.243805 |
| TCGA-FD-A62N-01A | -9.135408061 | 2.167080708 | -2048.338819 |
| TCGA-DK-AA75-01A | 7.433031769 | -1.876151145 | 1814.770119 |
| TCGA-XF-A9SL-01A | -1.886693675 | 5.640248524 | 1366.15715 |
| TCGA-XF-A9T4-01A | -6.83988392 | -0.509775053 | -2574.461471 |
| TCGA-XF-AAN2-01A | -2.427805859 | -2.181328337 | -1485.236175 |
| TCGA-BT-A2LD-01A | -5.922047273 | -0.373189625 | -1722.191697 |
| TCGA-ZF-A9R1-01A | 3.568626113 | 0.825631828 | 1502.01657 |
| TCGA-ZF-A9R9-01A | 2.174130612 | 6.348222856 | 3186.348147 |
| TCGA-DK-A6AV-01A | 4.163280231 | -0.779699634 | 1067.296307 |
| TCGA-XF-AAMY-01A | 2.406458039 | 4.586607144 | 2684.735802 |
| TCGA-GU-A763-01A | 8.669915538 | -4.419969089 | 1260.366101 |
| TCGA-FD-A3B7-01A | -11.270928 | 1.437472847 | -2951.644954 |
| TCGA-FD-A3NA-01A | 1.700639518 | 2.255781983 | 1602.475138 |
| TCGA-CF-A47T-01A | 6.599772616 | -1.316106303 | 1775.6585 |
| TCGA-BT-A0S7-01A | -6.384858438 | -3.388619146 | -2318.89946 |
| TCGA-GC-A3BM-01A | 3.314081623 | -4.175740309 | -244.4740073 |
| TCGA-GD-A3OP-01A | 5.579838049 | 3.913692896 | 3708.341046 |
| TCGA-SY-A9G5-01A | -4.783501838 | 4.117199938 | -234.7311423 |
| TCGA-KQ-A41S-01A | 1.383152825 | 3.995354641 | 1830.077334 |
| TCGA-4Z-AA7M-01A | 4.967040615 | 2.162546797 | 2415.077452 |
| TCGA-FD-A3SJ-01A | 3.95427097 | 3.864048999 | 2931.893084 |
| TCGA-E7-A677-01A | 3.925298561 | -0.194187189 | 1245.248849 |
| TCGA-UY-A8OD-01A | -0.414289651 | 4.975210281 | 1623.030493 |
| TCGA-DK-AA6L-01A | 0.640600198 | 0.964966383 | 553.5316756 |
| TCGA-GD-A2C5-01A | 5.110187613 | 4.009931966 | 3462.166267 |
| TCGA-K4-A4AC-01A | -9.78327215 | -3.980496558 | -3769.083103 |
| TCGA-FD-A62O-01A | 5.902440694 | 2.071566446 | 2955.863943 |
| TCGA-BT-A2LB-01A | 0.361832984 | 4.165760921 | 1790.563756 |
| TCGA-FD-A3N5-01A | -9.371759936 | -4.015506944 | -4198.400823 |
| TCGA-G2-A3VY-01A | 9.589476441 | -2.010708348 | 2247.357455 |
| TCGA-DK-A1AA-01A | 3.979343545 | -3.143741426 | 254.0080183 |
| TCGA-XF-A9SY-01A | -11.70820594 | 1.084450619 | -2955.483957 |
| TCGA-DK-AA6Q-01A | -6.149656246 | -7.194872921 | -3221.949099 |
| TCGA-G2-A2EL-01A | -1.487208628 | -6.389954853 | -1359.932878 |
| TCGA-E7-A6ME-01A | 7.103281452 | 0.87730929 | 2771.481315 |
| TCGA-HQ-A2OF-01A | 0.472308411 | -4.630263462 | -925.3524241 |
| TCGA-FD-A6TF-01A | -6.139018163 | 2.228891502 | -1273.114618 |
| TCGA-FD-A3SP-01A | -9.76875468 | 4.217194318 | -1626.831385 |
| TCGA-BT-A20P-01A | 6.994864144 | -1.175923915 | 1826.679454 |
| TCGA-GV-A40G-01A | 8.628337481 | -0.983764515 | 2377.929047 |
| TCGA-CF-A47S-01A | 10.03059746 | -1.791256433 | 2697.989575 |
| TCGA-XF-A9T2-01A | -5.200258678 | 1.26873764 | -1155.000735 |
| TCGA-CF-A9FH-01A | 9.955052599 | -2.693728126 | 2366.115284 |
| TCGA-ZF-AA4X-01A | 9.721451847 | -2.44160286 | 2322.807113 |
| TCGA-XF-AAMQ-01A | 2.886925131 | -1.005252218 | 571.1172133 |
| TCGA-CF-A3MH-01A | 5.828583646 | -3.065254804 | 803.6076523 |
| TCGA-DK-AA6W-01A | -3.374998304 | -3.005934207 | -1614.604819 |
| TCGA-KQ-A41O-01A | 9.595046836 | -3.350438277 | 1814.134794 |
| TCGA-FJ-A3Z7-01A | 4.551595369 | 1.869785991 | 2064.370463 |
| TCGA-S5-AA26-01A | 6.978489335 | -1.591644687 | 1828.955475 |
| TCGA-FD-A3B4-01A | -12.44923388 | -1.186298978 | -4305.918707 |
| TCGA-GC-A3OO-01A | 0.554066297 | 3.139198394 | 1354.71901 |
| TCGA-XF-A9SP-01A | -1.615421094 | 5.857289666 | 1609.579072 |
| TCGA-FD-A3SL-01A | 0.333521255 | 5.950078507 | 2335.090939 |
| TCGA-FD-A6TB-01A | -3.239308455 | 2.85350573 | -123.754888 |
| TCGA-FD-A6TA-01A | 2.315113396 | 4.83829166 | 2705.121043 |
| TCGA-XF-A8HB-01A | 2.325613272 | -5.405203426 | -768.1839082 |
| TCGA-UY-A8OB-01A | -7.580379911 | -2.487596922 | -2678.96341 |
| TCGA-GU-A767-01A | 5.696096255 | 2.649561945 | 3237.368015 |
| TCGA-FD-A43U-01A | 1.200867555 | 6.61457424 | 3212.043907 |
| TCGA-DK-AA6T-01A | -1.341410754 | -2.215222413 | -805.0491366 |
| TCGA-5N-A9KI-01A | -0.246192177 | 2.619240197 | 736.8719665 |
| TCGA-XF-A8HF-01A | 3.421904995 | 2.878547392 | 2128.832262 |
| TCGA-UY-A78L-01A | -6.349973689 | 0.129572596 | -2000.887835 |
| TCGA-FD-A5C0-01A | 0.272597599 | 1.54047705 | 592.5348527 |
| TCGA-UY-A9PE-01A | 3.544996323 | 3.407695781 | 2513.776358 |
| TCGA-K4-A6FZ-01A | -0.838612227 | -2.656077167 | -1319.943019 |
| TCGA-DK-A3IK-01A | 4.981713111 | 3.787313865 | 3427.849751 |
| TCGA-LC-A66R-01A | -7.288069245 | -2.063793812 | -2855.03919 |
| TCGA-GU-A762-01A | -9.704628404 | 0.256664667 | -2688.3197 |
| TCGA-4Z-AA7O-01A | 3.057653453 | -0.023198485 | 1105.798255 |
| TCGA-DK-A6B5-01A | -1.841710624 | -1.174475551 | -941.6266184 |
| TCGA-C4-A0F0-01A | -12.70913054 | -2.916284084 | -3897.852721 |
| TCGA-UY-A78N-01A | 4.24072275 | 1.47623594 | 1895.880402 |
| TCGA-G2-AA3F-01A | 9.197200554 | 1.419033308 | 3850.726534 |
| TCGA-ZF-AA53-01A | -10.43219299 | 0.255697406 | -3252.301452 |
| TCGA-LT-A8JT-01A | 9.355869845 | -1.249900989 | 2727.976182 |
| TCGA-XF-A8HI-01A | 7.791578908 | -2.047519297 | 1934.178569 |
| TCGA-FJ-A3ZE-01A | 4.769538471 | -3.860440401 | 243.1601386 |
| TCGA-FD-A3SM-01A | -2.205905307 | 3.60486771 | 444.6207384 |
| TCGA-DK-AA6X-01A | 2.066436193 | 0.655072229 | 857.2419513 |
| TCGA-XF-A9SJ-01A | -0.92867136 | 3.870667711 | 985.8002096 |
| TCGA-CF-A7I0-01A | 6.98829912 | 2.410308423 | 3428.952401 |
| TCGA-GD-A3OQ-01A | -2.573553242 | -0.419673462 | -1084.863066 |
| TCGA-CU-A0YO-01A | 1.078336458 | 0.112672885 | 392.6979462 |
| TCGA-DK-AA71-01A | 4.856130525 | -0.182965291 | 1565.936224 |
| TCGA-CF-A9FL-01A | 3.058002156 | 2.233365693 | 1891.713495 |
| TCGA-ZF-AA5N-01A | -2.60541581 | -2.462999028 | -1615.816548 |
| TCGA-UY-A9PF-01A | 5.526821225 | 0.971572545 | 2220.598859 |
| TCGA-UY-A78P-01A | -5.645121026 | -1.460951969 | -2276.934208 |
| TCGA-CF-A1HR-01A | 4.748377843 | 0.539535819 | 1794.57286 |
| TCGA-DK-A3IN-01A | -5.001302153 | 2.893920793 | -767.5892283 |
| TCGA-UY-A9PH-01A | 4.613354054 | 2.890455659 | 2940.732171 |
| TCGA-GD-A76B-01A | 7.266756083 | -0.200069085 | 2500.032872 |
| TCGA-ZF-AA58-01A | -10.12553363 | 1.647324074 | -2268.604724 |
| TCGA-ZF-AA4V-01A | -4.941503161 | 1.087191061 | -1223.682424 |
| TCGA-DK-AA6R-01A | -9.031759885 | -3.011853279 | -3990.889432 |
| TCGA-FT-A61P-01A | -5.500117717 | 2.929517386 | -825.6878556 |
| TCGA-2F-A9KQ-01A | 7.97613963 | 0.536358157 | 2956.16127 |
| TCGA-K4-A54R-01A | -0.577021561 | 4.117956983 | 1304.810569 |
| TCGA-FD-A62S-01A | -10.71095818 | 2.493339137 | -2644.231858 |
| TCGA-ZF-A9R3-01A | 8.153014943 | 0.497951794 | 2985.591958 |
| TCGA-BT-A42F-01A | -7.596854356 | -3.836447363 | -2831.747443 |
| TCGA-K4-A83P-01A | -0.094930953 | 4.704667067 | 1704.872835 |
| TCGA-K4-A5RJ-01A | -6.283519131 | -1.669996411 | -1688.856944 |
| TCGA-XF-A9SX-01A | -6.806712692 | 3.773805859 | -897.6901642 |
| TCGA-KQ-A41N-01A | 5.148638556 | -0.414871985 | 1441.580808 |
| TCGA-DK-A1AE-01A | -4.624273021 | -5.594119644 | -2220.83514 |
| TCGA-DK-A3WX-01A | -10.50195898 | -0.607135334 | -3459.289946 |
| TCGA-DK-A3WW-01A | -9.694619314 | -4.880372681 | -3691.36309 |
| TCGA-XF-A9SK-01A | -0.948840206 | 5.638434021 | 1779.983847 |
| TCGA-KQ-A41R-01A | 9.643111363 | -0.255314377 | 3228.823293 |
| TCGA-CF-A3MG-01A | 8.056980646 | -3.378000738 | 1393.483234 |
| TCGA-CU-A0YR-01A | 1.453739352 | 1.699701177 | 1041.420824 |
| TCGA-UY-A78M-01A | 1.518006826 | 3.941551989 | 2130.847275 |
| TCGA-CF-A5UA-01A | 5.453528985 | -2.413799157 | 844.484854 |
| TCGA-ZF-A9RN-01A | -2.581425026 | -4.565090935 | -1354.432018 |
| TCGA-4Z-AA81-01A | -2.272315804 | -3.447465237 | -1649.907253 |
| TCGA-E7-A8O8-01A | 6.783819343 | -2.620858056 | 1254.47135 |
| TCGA-GC-A3RB-01A | 2.189075365 | -2.183039793 | 1.797087521 |
| TCGA-BT-A20O-01A | -9.411591724 | 0.458028888 | -1935.057131 |
| TCGA-G2-A2ES-01A | -9.631254336 | -4.065724521 | -4123.605456 |
| TCGA-DK-AA6P-01A | 9.717601578 | -0.916959443 | 3029.571662 |
| TCGA-FD-A43P-01A | 3.664305285 | 2.699347013 | 2237.897613 |
| TCGA-2F-A9KT-01A | -1.369840283 | -0.142094954 | -465.1945784 |
| TCGA-CF-A9FF-01A | 7.983737989 | 1.733607286 | 3659.642279 |
| TCGA-UY-A9PA-01A | 3.238651056 | -2.436913882 | 278.2712126 |
| TCGA-E7-A7DU-01A | 8.920113522 | -1.743359775 | 2379.700767 |
| TCGA-2F-A9KW-01A | -0.635479304 | 0.817404182 | 49.7204552 |
| TCGA-4Z-AA87-01A | 6.136349371 | 1.255857382 | 2494.370828 |
| TCGA-GU-A42Q-01A | -8.842332663 | -4.507695643 | -3512.254282 |
| TCGA-E7-A97P-01A | -8.349948147 | -0.823848166 | -3111.493311 |
| TCGA-XF-AAMG-01A | 2.406428887 | 3.263175197 | 1955.49533 |
| TCGA-BT-A3PH-01A | 3.81801087 | -0.885963593 | 886.9678911 |
| TCGA-ZF-AA4R-01A | -3.352223389 | 4.924216519 | 594.3305794 |
| TCGA-ZF-AA5P-01A | -1.130504871 | 5.13437682 | 1459.68754 |
| TCGA-DK-A3X2-01A | -0.59854695 | -5.733168333 | -1607.783248 |
| TCGA-YC-A8S6-01A | 1.353468638 | 2.242380784 | 1391.555898 |
| TCGA-GC-A3RC-01A | -10.83920013 | -3.967161662 | -4062.239998 |
| TCGA-BL-A0C8-01A | 5.902028576 | 1.204216882 | 2405.947182 |
| TCGA-GU-AATP-01A | 2.360287677 | 6.054845121 | 3247.779075 |
| TCGA-CF-A1HS-01A | -8.084440481 | -3.16084253 | -3677.759392 |
| TCGA-ZF-AA4U-01A | 5.474086249 | -5.58018832 | -29.28172332 |
| TCGA-GV-A3QF-01A | 9.991428903 | -0.832308565 | 2946.185312 |
| TCGA-GC-A4ZW-01A | 3.515208659 | -3.423618666 | 23.02359695 |
| TCGA-ZF-A9RD-01A | -10.58577077 | -4.359490486 | -4604.218508 |
| TCGA-GU-A766-01A | -9.053957877 | -3.651124568 | -3575.807477 |
| TCGA-G2-A2EF-01A | -8.658313937 | -3.28892593 | -2901.069352 |
| TCGA-FD-A5BY-01A | -5.734989972 | 0.983124191 | -1632.195985 |
| TCGA-CF-A8HX-01A | 8.793218146 | -0.752001618 | 2561.290798 |
| TCGA-GV-A6ZA-01A | 9.040854032 | -0.884756405 | 2878.022282 |
| TCGA-DK-A1A5-01A | -4.03468692 | 4.039505094 | 1.623081971 |
| TCGA-4Z-AA7N-01A | 0.124480957 | 3.46077836 | 1313.327543 |
| TCGA-GC-A3RD-01A | 3.999495923 | 0.063958887 | 1395.769982 |
| TCGA-G2-A3IE-01A | 9.16532194 | -1.278802962 | 2644.135799 |
| TCGA-FD-A5BZ-01A | -2.1167863 | 6.676235691 | 1820.292861 |
| TCGA-BT-A3PJ-01A | -9.113508777 | -2.445018799 | -2923.25835 |
| TCGA-CU-A72E-01A | -3.540140376 | -1.151941944 | -1519.694258 |
| TCGA-ZF-A9RM-01A | 11.31554116 | -2.502803191 | 2861.451131 |
| TCGA-FD-A6TH-01A | -6.169643062 | 1.015142718 | -1430.523744 |
| TCGA-GD-A3OS-01A | -8.913626101 | 0.926490234 | -2164.841522 |
| TCGA-DK-A3IS-01A | 5.886490098 | -4.273239639 | 468.4950275 |
| TCGA-K4-A3WS-01A | -0.435821217 | 6.709841076 | 2486.193074 |
| TCGA-4Z-AA7R-01A | 4.633432653 | 0.736927793 | 1725.266195 |
| TCGA-DK-A6B1-01A | 9.436210978 | 1.391743704 | 3829.200903 |
| TCGA-GV-A3QI-01A | 5.563305823 | -1.801584906 | 1107.906702 |
| TCGA-DK-A6AW-01A | 2.963143885 | -2.734164513 | 60.73995695 |
| TCGA-BT-A20W-01A | 6.278055376 | 2.219162216 | 3107.406283 |
| TCGA-DK-A3IU-01A | -8.5491518 | 1.600867054 | -2053.7194 |
| TCGA-GC-A3YS-01A | -8.590030496 | 1.502777198 | -2152.599168 |
| TCGA-4Z-AA7Q-01A | -1.238042296 | -2.612170574 | -1047.197816 |
| TCGA-LT-A5Z6-01A | 10.39255477 | -0.646677187 | 3378.118911 |
| TCGA-GC-A3I6-01A | -4.89565297 | -2.001607394 | -2240.800409 |
| TCGA-E7-A5KF-01A | 11.68487017 | -4.274433593 | 2306.108382 |
| TCGA-DK-A3IM-01A | -9.692788779 | -6.685943428 | -4283.470567 |
| TCGA-CF-A47X-01A | 11.09327481 | -2.0242814 | 3025.649053 |
| TCGA-BT-A2LA-01A | -3.440404059 | -2.480107589 | -1114.929495 |
| TCGA-FD-A6TD-01A | -6.490351095 | 1.204410697 | -1661.322091 |
| TCGA-XF-AAMX-01A | 5.36159315 | 3.29092191 | 3189.958849 |
| TCGA-ZF-A9R5-01A | 7.489978397 | -4.819443551 | 749.1737198 |
| TCGA-XF-A8HC-01A | 11.06581334 | -1.317405996 | 3335.554919 |
| TCGA-ZF-A9RF-01A | -4.394975161 | -1.426357095 | -1345.041144 |
| TCGA-H4-A2HO-01A | 7.600583112 | 0.793366419 | 2833.033545 |
| TCGA-BT-A42E-01A | -8.791065501 | -3.403774881 | -3316.223588 |
| TCGA-FD-A5BU-01A | -11.85505163 | -1.512047601 | -4252.687232 |
| TCGA-XF-A9T6-01A | -6.493162239 | -2.508068737 | -2547.218349 |
| TCGA-ZF-AA4T-01A | 7.258592851 | 0.104709739 | 2221.396023 |
| TCGA-FD-A3B6-01A | -8.322420496 | -0.50177278 | -2372.888672 |
| TCGA-4Z-AA80-01A | 8.172009858 | -2.468557051 | 1696.172317 |
| TCGA-XF-A9SW-01A | -5.541381291 | 5.061398514 | -159.0216712 |
| TCGA-E7-A5KE-01A | 8.41038689 | -3.572851794 | 1362.367327 |
| TCGA-DK-AA6S-01A | -4.296324375 | 3.536087111 | -255.703807 |
| TCGA-FD-A5BR-01A | 1.933207466 | 2.332382189 | 1685.136914 |
| TCGA-FD-A6TC-01A | -1.287716213 | 5.061687737 | 1339.841234 |
| TCGA-XF-AAN1-01A | 8.073145418 | -1.051557067 | 2413.915911 |
| TCGA-DK-A3IV-01A | 7.019957014 | -1.867581294 | 1581.01312 |
| TCGA-PQ-A6FN-01A | -8.823824667 | -1.205604319 | -3325.553094 |
| TCGA-K4-A5RI-01A | -4.923516846 | -2.097753984 | -2067.43552 |
| TCGA-ZF-A9R7-01A | 5.990403685 | -0.62384422 | 1857.801052 |
| TCGA-XF-A9SM-01A | -9.314993178 | 0.442849516 | -2649.985149 |
| TCGA-FD-A6TE-01A | 5.984102576 | -2.122971869 | 1180.134611 |
| TCGA-XF-AAME-01A | -8.655156566 | 1.526119641 | -2626.829838 |
| TCGA-BT-A42C-01A | 5.963092773 | -2.183962169 | 1194.974012 |
| TCGA-CF-A47Y-01A | 8.617990493 | -2.484533795 | 1860.479856 |
| TCGA-E7-A519-01A | 6.60075238 | -0.265503316 | 2042.121937 |
| TCGA-FD-A43Y-01A | -9.667480566 | -1.321064007 | -3415.395676 |
| TCGA-BL-A5ZZ-01A | -9.789338683 | 1.997377177 | -2308.178729 |
| TCGA-CF-A3MF-01A | 8.285470835 | -2.591299253 | 1602.635768 |
| TCGA-FD-A3B8-01A | -10.07808206 | 2.21020515 | -2305.361517 |
| TCGA-DK-A1A7-01A | 6.465762926 | -1.136555515 | 1729.26823 |
| TCGA-DK-A1A6-01A | 3.137606491 | -0.036094753 | 899.4268666 |
| TCGA-DK-A6B2-01A | 1.666646869 | 4.757087417 | 2329.269132 |
| TCGA-DK-AA74-01A | -9.94186704 | 2.444443294 | -2455.517565 |
| TCGA-ZF-AA4N-01A | -11.00404205 | -3.838374089 | -3665.260141 |
| TCGA-BT-A0YX-01A | -5.952702171 | -5.794643997 | -3058.113432 |
| TCGA-R3-A69X-01A | 0.709614266 | 4.194838677 | 1770.166136 |
| TCGA-XF-AAMW-01A | -9.014683923 | -1.094475983 | -3246.735814 |
| TCGA-C4-A0EZ-01A | -3.585078396 | -3.056801658 | -1546.785077 |
| TCGA-E7-A678-01A | 9.381362145 | -4.446083986 | 1448.655184 |
| TCGA-G2-A3IB-01A | -8.189464141 | -6.04355019 | -4433.535847 |
| TCGA-DK-A3IT-01A | 0.874665723 | 4.048470522 | 1776.007403 |
| TCGA-MV-A51V-01A | 7.89209452 | 0.807618223 | 3044.411027 |
| TCGA-UY-A78O-01A | 7.990028491 | 1.840783081 | 3647.785868 |
| TCGA-FD-A6TK-01A | -9.542482242 | 2.710422813 | -2186.213642 |
| TCGA-4Z-AA83-01A | 4.513162977 | 0.29297713 | 1645.209451 |
| TCGA-DK-A2I6-01A | -5.865571397 | -0.943977697 | -1730.363481 |
| TCGA-XF-AAN4-01A | -9.318621581 | 2.391194811 | -1930.004418 |
| TCGA-CF-A27C-01A | 9.268089244 | -3.8612699 | 1581.960298 |
| TCGA-5N-A9KM-01A | 2.307565063 | 2.712060977 | 1646.874524 |
| TCGA-BT-A20U-01A | -4.863313309 | -1.721346779 | -2109.784775 |
| TCGA-YC-A9TC-01A | -3.374802228 | -2.61559217 | -1268.219822 |
| TCGA-E7-A6MD-01A | 1.591159204 | 2.753907073 | 1543.524447 |
| TCGA-K4-A5RH-01A | -9.294850829 | 1.29495703 | -1846.457853 |
| TCGA-XF-AAMH-01A | -2.952810642 | -1.699421643 | -1361.398747 |
| TCGA-DK-A1AB-01A | -8.583196139 | 1.492919547 | -2313.20877 |
| TCGA-E5-A4TZ-01A | -0.946017803 | -6.695621042 | -1977.177964 |
| TCGA-FD-A43X-01A | 9.497938936 | -2.181090583 | 2368.531435 |
| TCGA-XF-A9SU-01A | 0.920153877 | 5.338619099 | 2484.846643 |
| TCGA-BT-A20N-01A | -0.621146208 | -0.557054221 | -356.4468884 |
| TCGA-BT-A20J-01A | -4.674441684 | -2.071660428 | -1596.346664 |
| TCGA-E7-A4IJ-01A | 8.104509498 | -0.671861854 | 2463.641374 |
| TCGA-CF-A47W-01A | 8.919115581 | -2.299541083 | 2079.890275 |
| TCGA-XF-A9SV-01A | 2.671335831 | 4.681199323 | 2777.129626 |
| TCGA-K4-AAQO-01A | 1.531348356 | 4.647175566 | 2370.30723 |
| TCGA-CU-A3YL-01A | 8.445387101 | 1.019059709 | 3401.611774 |
| TCGA-DK-A2I2-01A | -11.04002634 | -1.167550624 | -3784.984986 |
| TCGA-4Z-AA84-01A | -4.276417735 | -0.781941593 | -1297.925058 |
| TCGA-DK-A1AF-01A | -3.161253472 | 3.881241162 | 294.7726423 |
| TCGA-E5-A4U1-01A | 12.53074504 | -2.024730245 | 3578.887421 |
| TCGA-BT-A20Q-01A | -0.113087669 | 3.600879221 | 1233.400228 |
| TCGA-ZF-A9R4-01A | 8.305033562 | -3.006793596 | 1651.063478 |
| TCGA-KQ-A41Q-01A | 6.42611586 | 1.837606729 | 2713.646549 |
| TCGA-E7-A8O7-01A | 8.560673701 | -0.437026022 | 2849.490008 |
| TCGA-ZF-AA4W-01A | -9.362126551 | -6.118985778 | -4393.227356 |
| TCGA-XF-AAN7-01A | -6.523938471 | 1.692017082 | -1097.216606 |
| TCGA-XF-A8HH-01A | 4.34714962 | 5.280435948 | 3736.751755 |
| TCGA-FD-A5BX-01A | -9.228964634 | 3.836680189 | -1472.77695 |
| TCGA-ZF-A9RE-01A | -0.987746544 | -7.768077157 | -2505.105258 |
| TCGA-FT-A3EE-01A | -1.456016783 | -0.515710554 | -600.7784895 |
| TCGA-XF-A9T5-01A | -6.217312759 | -1.996588503 | -2035.556501 |
| TCGA-XF-AAML-01A | 8.204409735 | -3.050474474 | 1535.730406 |
| TCGA-BT-A3PK-01A | -9.408019631 | -1.223842551 | -3347.086848 |
| TCGA-CF-A3MI-01A | 6.303006166 | -1.894474741 | 1311.617432 |
| TCGA-E7-A541-01A | 4.063941554 | -0.270198913 | 1129.746238 |
| TCGA-ZF-AA54-01A | -10.05540439 | 1.392490415 | -1995.098405 |
| TCGA-XF-AAMZ-01A | 6.663791252 | 1.045813046 | 2671.801707 |
| TCGA-CF-A8HY-01A | 4.975269676 | -3.541744107 | 397.942209 |
| TCGA-GV-A3QG-01A | -3.979120045 | 4.184774192 | 65.76201288 |
| TCGA-DK-A6B6-01A | 10.82926588 | -1.823438161 | 2972.151979 |
| TCGA-DK-A6B0-01A | 8.885892946 | -3.16654853 | 1750.978721 |
| TCGA-E7-A97Q-01A | 6.537442568 | 4.412978293 | 4457.196176 |
| TCGA-2F-A9KP-01A | 8.054266621 | -0.849240794 | 2348.122338 |
| TCGA-FD-A62P-01A | -4.988448319 | 1.375963728 | -1225.409354 |
| TCGA-XF-A8HG-01A | 10.39158221 | -2.72669759 | 2277.719196 |
| TCGA-4Z-AA86-01A | -9.643399892 | 1.199563319 | -2675.550997 |
| TCGA-GD-A6C6-01A | 5.276727305 | 0.337965843 | 1730.205362 |
| TCGA-G2-A2EJ-01A | -9.59162492 | -6.994100685 | -4724.531579 |
| TCGA-UY-A9PD-01A | 3.551159055 | 5.216203432 | 3377.106901 |
| TCGA-S5-A6DX-01A | 2.838035225 | 2.827274671 | 2015.396698 |
| TCGA-FD-A3SQ-01A | -0.695249734 | 5.631997759 | 1852.216649 |
| TCGA-DK-AA77-01A | 6.731451875 | -3.251716127 | 1102.115797 |
| TCGA-BL-A13I-01A | -8.043010362 | -0.593931498 | -1698.702986 |
| TCGA-K4-A6MB-01A | 1.923458236 | 2.873118302 | 1909.711607 |
| TCGA-ZF-AA51-01A | 3.941470799 | 2.941223974 | 2384.582451 |
| TCGA-E7-A7PW-01A | 9.630816466 | -1.323674745 | 2811.542634 |
| TCGA-E7-A85H-01A | 4.612907693 | 2.935326246 | 2675.428754 |
| TCGA-XF-AAMR-01A | -1.973145954 | 3.443679499 | 503.342989 |
| TCGA-FD-A6TG-01A | 0.694623442 | 5.101807163 | 2072.842489 |
| TCGA-2F-A9KO-01A | 3.001056915 | 1.769759133 | 1779.574767 |
| TCGA-HQ-A5ND-01A | -4.780750717 | -4.873342225 | -2451.421899 |
| TCGA-G2-AA3B-01A | 10.33876898 | -1.43390137 | 3124.173196 |
| TCGA-PQ-A6FI-01A | -10.48936918 | -5.838510132 | -4277.145899 |
| TCGA-C4-A0F7-01A | -6.56770332 | -5.30571746 | -3077.132107 |
| TCGA-FD-A5BS-01A | -7.763251643 | 3.328803471 | -1225.854572 |
| TCGA-GV-A3JX-01A | 3.835971211 | -1.491454892 | 757.2505967 |
| TCGA-XF-AAMT-01A | -8.721691043 | 1.825624474 | -2323.294789 |
| TCGA-XF-A9T8-01A | -9.152252114 | -3.388636144 | -3629.007922 |
| TCGA-4Z-AA7S-01A | 7.780752492 | 0.19738177 | 2688.828118 |
| TCGA-XF-A9SH-01A | 6.097045314 | 0.228118285 | 2114.192929 |
| TCGA-DK-A1AG-01A | 6.249028419 | -3.582722426 | 854.3121676 |
| TCGA-2F-A9KR-01A | 2.4368676 | -2.551761305 | -38.64491261 |
| TCGA-DK-AA6U-01A | 7.033205359 | -1.50278359 | 1874.188507 |
| TCGA-C4-A0F6-01A | 1.477882078 | 1.403048587 | 963.9504046 |
| TCGA-CU-A0YN-01A | -8.219990969 | -1.862087546 | -3356.839519 |
| TCGA-FJ-A871-01A | -9.146807299 | 2.441729506 | -1771.336163 |
| TCGA-G2-A2EC-01A | 1.875996833 | 3.339605551 | 1825.530677 |
| TCGA-FD-A5BV-01A | 3.467704166 | 0.553763628 | 1348.188771 |
| TCGA-E7-A7XN-01A | -6.318858175 | -6.264756022 | -3310.783604 |
| TCGA-GU-A764-01A | -7.019508403 | 1.280028623 | -1847.89394 |
| TCGA-XF-AAN8-01A | -10.11436364 | 4.033647561 | -1625.37406 |
| TCGA-XF-A9SZ-01A | -0.943630052 | 5.79776011 | 1789.970459 |
| TCGA-GU-AATO-01A | 0.951662736 | 2.198759733 | 1003.54546 |
| TCGA-ZF-A9RC-01A | 0.501803584 | 5.48609937 | 2246.876635 |
| TCGA-ZF-A9R0-01A | 6.280431314 | 3.944861625 | 3982.038805 |
| TCGA-KQ-A41P-01A | 3.708070593 | 3.709491339 | 2863.758004 |
| TCGA-4Z-AA7W-01A | -5.715610208 | -1.574640513 | -1973.916492 |
| TCGA-HQ-A5NE-01A | -3.578394367 | 0.531385194 | -1127.073412 |
| TCGA-CU-A3KJ-01A | -8.5424545 | -4.597458229 | -3065.708707 |
| TCGA-UY-A8OC-01A | -10.56178485 | 1.95571703 | -2852.992766 |
| TCGA-E7-A3Y1-01A | 6.748918372 | -2.190050551 | 1423.510174 |
| TCGA-XF-AAN5-01A | -7.414185077 | -3.069731893 | -2806.669134 |
| TCGA-G2-AA3D-01A | 7.583967789 | -1.974283438 | 1818.290464 |
| TCGA-GU-A42R-01A | 4.076082197 | -3.03454632 | 297.4417797 |
| TCGA-XF-A9SI-01A | -4.828651825 | 1.744466597 | -851.3595713 |
| TCGA-GV-A3JZ-01A | 3.623721893 | 2.83153206 | 2143.135656 |
