# Supplementary material for: RNA m5C methylation orchestrates BLCA progression via macrophage reprogramming
Source: J Cell Mol Med. 2023 Jul 5;27(16):2398–411. doi: 10.1111/jcmm.17826 (PMC10424284; doi:10.1111/jcmm.17826)
Supplement: Supplementary file 3 — Figure S3. [file JCMM-27-2398-s005.pdf]

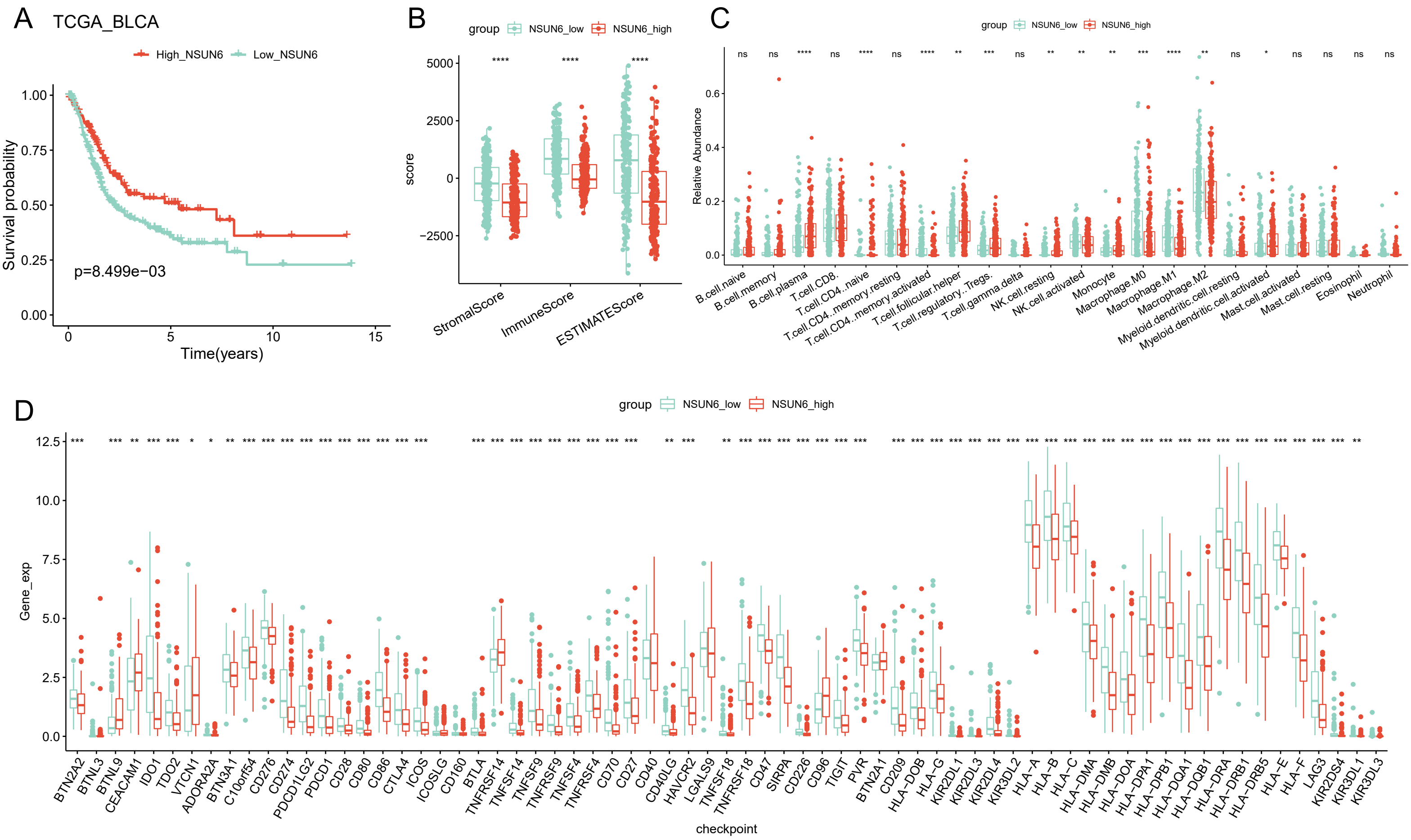
A
TCGA_BLCA
High_NSUN6
Low_NSUN6
Survival probability
1.00
0.75
0.50
0.25
0.00
p=8.499e−03
0
5
10
15
Time(years)
B
group
NSUN6_low
NSUN6_high
5000
2500
0
−2500
score
****
****
****
StromalScore
ImmuneScore
ESTIMATEScore
C
group
NSUN6_low
NSUN6_high
Relative Abundance
0.6
0.4
0.2
0.0
ns ns **** ns **** ns **** ** *** ns ** ** ** *** **** ** ns * ns ns ns ns
B.cell.naive
B.cell.memory
B.cell.plasma
T.cell.CD8.
T.cell.CD4..naive
T.cell.CD4..memory.resting
T.cell.CD4..memory.activated
T.cell.follicular.helper
T.cell.regulatory..Tregs.
T.cell.gamma.delta
NK.cell.resting
NK.cell.activated
Monocyte
Macrophage.M0
Macrophage.M1
Macrophage.M2
Myeloid.dendritic.cell.resting
Myeloid.dendritic.cell.activated
Mast.cell.activated
Mast.cell.resting
Eosinophil
Neutrophil
D
group
NSUN6_low
NSUN6_high
Gene_exp
12.5
10.0
7.5
5.0
2.5
0.0
checkpoint
BTN2A2
BTNL3
BTNL9
CEACAM1
IDO1
TDO2
VTCN1
ADORA2A
BTN3A1
C10orf54
CD276
CD274
PDCD1LG2
PDCD1
CD28
CD80
CD86
CTLA4
ICOS
ICOSLG
CD160
BTLA
TNFRSF14
TNFSF14
TNFSF9
TNFRSF9
TNFSF4
TNFRSF4
CD70
CD27
CD40
CD40LG
HAVCR2
LGALS9
TNFSF18
TNFRSF18
CD47
SIRPA
CD226
CD96
TIGIT
PVR
BTN2A1
CD209
HLA−DOB
HLA−G
KIR2DL1
KIR2DL3
KIR2DL4
KIR3DL2
HLA−A
HLA−B
HLA−C
HLA−DMA
HLA−DMB
HLA−DOA
HLA−DPA1
HLA−DPB1
HLA−DQA1
HLA−DQB1
HLA−DRA
HLA−DRB1
HLA−DRB5
HLA−E
HLA−F
LAG3
KIR2DS4
KIR3DL1
KIR3DL3
